# Extracellular Matrix Analysis of Human Renal Arteries in Both Quiescent and Active Vascular State

**DOI:** 10.3390/ijms21113905

**Published:** 2020-05-30

**Authors:** Christian G.M. van Dijk, Laura Louzao-Martinez, Elise van Mulligen, Bart Boermans, Jeroen A.A. Demmers, Thierry P.P. van den Bosch, Marie-José Goumans, Dirk J. Duncker, Marianne C. Verhaar, Caroline Cheng

**Affiliations:** 1 Department of Nephrology and Hypertension, Division of Internal Medicine and Dermatology, University Medical Center Utrecht, PO Box 85500, 3508 GA Utrecht, The Netherlands; c.g.m.vandijk-4@umcutrecht.nl (C.G.M.v.D.); e.vanmulligen2@gmail.com (E.v.M.); boermans1990@gmail.com (B.B.); m.c.verhaar@umcutrecht.nl (M.C.V.); 2 Center for Proteomics, Erasmus Medical Center, 3015 CN Rotterdam, The Netherlands; l.louzao.martinez@outlook.com (L.L.-M.); j.demmers@erasmusmc.nl (J.A.A.D.); 3 Department of Pathology, Erasmus University Medical Center, 3015 CN Rotterdam, The Netherlands; t.vandenbosch@erasmusmc.nl; 4 Department of Cell and Chemical Biology, Leiden University Medical Center, PO Box 9600, 2300 RC Leiden, The Netherlands; m.j.t.h.goumans@lumc.nl; 5 Experimental Cardiology, Department of Cardiology, Thorax center, Erasmus MC, University Medical Center Rotterdam, PO Box 2040, 3000 CA Rotterdam, The Netherlands; d.duncker@erasmusmc.nl

**Keywords:** extracellular matrix, proteomics, EMILIN1, FBN1, vasculature

## Abstract

In vascular tissue engineering strategies, the addition of vascular-specific extracellular matrix (ECM) components may better mimic the in vivo microenvironment and potentially enhance cell–matrix interactions and subsequent tissue growth. For this purpose, the exact composition of the human vascular ECM first needs to be fully characterized. Most research has focused on characterizing ECM components in mature vascular tissue; however, the developing fetal ECM matches the active environment required in vascular tissue engineering more closely. Consequently, we characterized the ECM protein composition of active (fetal) and quiescent (mature) renal arteries using a proteome analysis of decellularized tissue. The obtained human fetal renal artery ECM proteome dataset contains higher levels of 15 ECM proteins versus the mature renal artery ECM proteome, whereas 16 ECM proteins showed higher levels in the mature tissue compared to fetal. Elastic ECM proteins EMILIN1 and FBN1 are significantly enriched in fetal renal arteries and are mainly produced by cells of mesenchymal origin. We functionally tested the role of EMILIN1 and FBN1 by anchoring the ECM secreted by vascular smooth muscle cells (SMCs) to glass coverslips. This ECM layer was depleted from either EMILIN1 or FBN1 by using siRNA targeting of the SMCs. Cultured endothelial cells (ECs) on this modified ECM layer showed alterations on the transcriptome level of multiple pathways, especially the Rho GTPase controlled pathways. However, no significant alterations in adhesion, migration or proliferation were observed when ECs were cultured on EMILIN1- or FNB1-deficient ECM. To conclude, the proteome analysis identified unique ECM proteins involved in the embryonic development of renal arteries. Alterations in transcriptome levels of ECs cultured on EMILIN1- or FBN1-deficient ECM showed that these candidate proteins could affect the endothelial (regenerative) response.

## 1. Introduction

The human vasculature mainly consists of two different cell types that have their own specific function. Along with the endothelial cells (ECs) that actually compose the blood vessels, providing main blood barrier function, mural cells also play an important role in maintaining vascular homeostasis. From these, pericytes associate with capillaries to help maintain barrier function, whereas vascular smooth muscle cells (SMCs) are found predominantly on larger arteries for biomechanical strength and elasticity. These cells create their own dynamic microenvironment for protection and stability by synthesizing and secreting extracellular matrix (ECM) components, including constituents of the basement membrane [1,2]. This matrix microenvironment consists of many components, such as collagens, proteoglycans, and glycoproteins, and forms a reservoir for encapsulated growth factors [3,4]. Thus, ECM proteins do not only provide structural and organizational stability for the surrounding cells, they are also responsible for a wide variety of biochemical cues. 

ECM remodeling is a critical step during vascular development. At the onset of angiogenesis, the basement membrane matrix is degraded to allow endothelial sprouting. As angiogenesis proceeds, the ECM is known to provide signals controlling EC migration, proliferation, survival, differentiation, shape, and invasion. Ultimately, through specific integrin-signaling pathways, the ECM controls the EC cytoskeleton to guide vascular morphogenesis towards a mature vascular network with functional lumens [5]. During this vascular response, different ECM cues are active. For example, ECs cultured on a collagen type 1 coating are stimulated to rearrange their cytoskeleton and cell junctions, inducing capillary morphogenesis. In contrast, culturing ECs on a laminin-1 coating inhibits this rearrangement [6]. These in vitro observations are in concordance with the laminin-rich basement membrane characteristics of in vivo mature vessels in a quiescent state [7]. This type of ECM component-driven response has also been described in (embryogenic) angiogenesis. Pro-angiogenic cues stimulate the breakdown of the vascular basement membrane (consisting mainly of laminins and collagen type 4) that exposes ECs to collagen type 1, which is abundant between cells in tissues. This triggers EC proliferation and vascular sprouting, while formation of a new basement membrane during neovessel maturation brings the endothelium back into the quiescent state [5]. Mapping the differences in ECM composition during embryonic vessel development and comparing these with mature stable vessels could help to shed light on the effect of different ECM components on the regenerative capacity of vascular cells. 

Applied technologies, such as vascular tissue engineering and regenerative medicine, could benefit from mimicking the vascular-specific ECM that is specifically active during development. At the moment, the regenerative cardiovascular research field uses multiple materials (synthetic and biological) to recreate functional grafts. For example, polymer scaffolds are being used to mimic blood vessels and heart valves in combination with ex vivo or in situ tissue engineering (TE) [8,9]. For in situ TE in particular, achieving a balance between the biodegradability of a scaffold and how fast cells are attracted remains a significant challenge. The addition of vascular-specific ECM components to these scaffolds might better mimic the in vivo microenvironment and enhance cell–matrix interactions and subsequent cell survival and tissue growth. In order to create such a scaffold, the components of the human vascular ECM first need to be fully characterized. 

Mass spectrometry-based approaches have been used to characterize ECM compositions of various tissues [3,10]. To unravel the vascular ECM, previous work mainly focused on mapping the ECM components in healthy, mature tissue in comparison with diseased tissue, rather than the changes in ECM during vascular development [11,12]. However, the fetal microenvironment is more dynamic and active compared to the matured ECM in order to cope with tissue morphogenesis and growth, which matches the environment needed in tissue scaffolds.

The aim of the present study was therefore to identify ECM components in vascular tissue during fetal development compared to the mature condition. By using proteomics, we created a catalogue of ECM proteins present in fetal renal arteries (in active, developmental state) and mature renal arteries (in stable, quiescent state). From this unique vascular ECM proteome, we selected two elastic components, Elastin Microfibril Interfacer 1 (EMILIN1) and fibrillin-1 (FBN1), based on their abundance in the fetal renal artery, as candidates for further investigation. Both EMILIN1 and FBN1 are glycoproteins located at elastic fibers and microfibrils [13,14]. Next to structural functions, EMILIN1 is known as a ligand for integrins, connecting cells with the surrounding ECM [15,16]. Many cell types need this integrin–EMILIN1 interaction for adhesion, migration, and proliferation [17,18,19,20]. Deficiency of either EMILIN1 or FBN1 in mice leads to aortic valve disease and vascular abnormalities, respectively [21,22]. Mutations in the FBN1 gene cause Marfan syndrome, a genetic connective tissue disorder characterized by aortic aneurysms and dissections [23]. Furthermore, both EMILIN1 and FBN1 seem to play an important role in maintaining vascular homeostasis [24]. Therefore, we investigated the role of both ECM components in vascular regeneration. 

## 2. Results

### 2.1. Enrichment of ECM Proteins in Vascular Tissue Prior to LC-MS/MS

**Figure 1:**
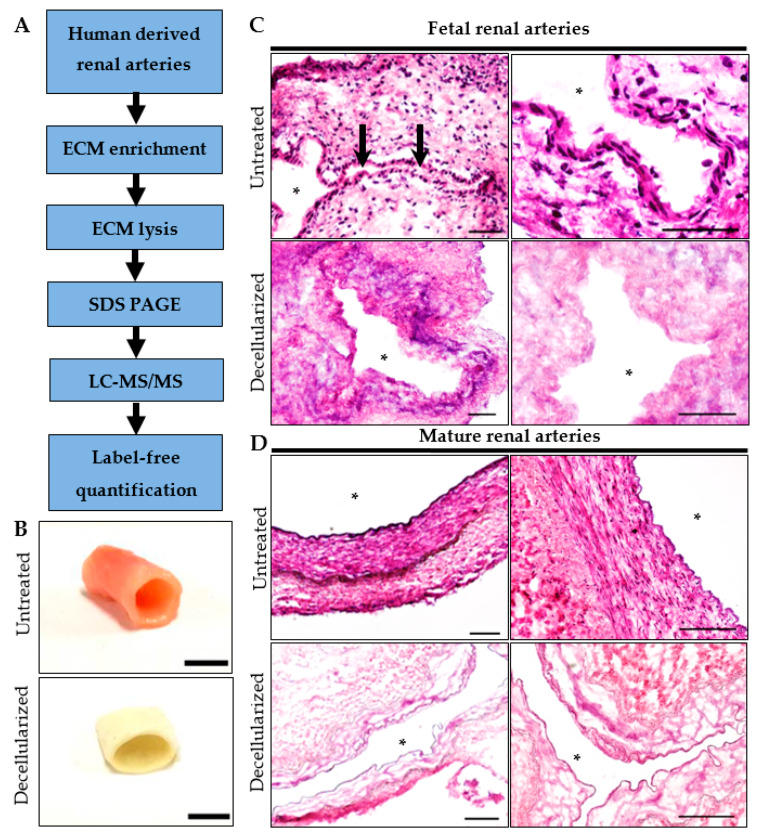
Proteomic workflow for extracellular matrix (ECM)-enriched vascular samples. (**A**) After decellularization of the human tissue, ECM-enriched samples were lysed and separated with SDS-PAGE prior to LC-MS/MS with label-free quantification. (**B**) Mature human renal arteries, before and after decellularization. Scale bar represents 1 cm. Hematoxylin and eosin staining for validation of decellularization procedure in (**C**) fetal human renal artery and (**D**) mature human renal artery. Black arrows indicate lumen. Open lumen is indicated with an asterix. Representative of 3 experiments. Scale bar represents 200 µm.

Healthy fetal and mature renal arteries were decellularized to enrich for ECM components (Figure 1A). The white appearance of the tissue after decellularization indicated loss of cells (Figure 1B). HE-staining confirmed complete decellularization, visualized by the absence of nuclei while maintaining the ECM architecture (Figure 1C,D). Proteins detected by LC-MS/MS in at least two pooled groups were used for further analysis (Figure S1A,B). Detected fetal and mature proteins were categorized by cross-referencing with the Human Matrisome Project [3,10]. 

Of all proteins detected, 39% of the fetal and 35% of the mature samples were composed of matrisome components. The majority forms part of the matrisome core proteins, subdivided by glycoproteins, collagens, and proteoglycans. A quarter of the matrisome proteins detected were allocated to matrisome-associated proteins, such as ECM-affiliated proteins, ECM regulators, and growth factors. There was only a subtle difference between the number of detected proteins of fetal and mature human arteries (Table 1). 

### 2.2. Matrisome Protein Expression Differs between Human Fetal and Mature Renal Arteries

Although there was almost no difference in the total number of different ECM proteins detected, relative protein quantification revealed a difference in protein abundance between fetal and mature samples. Mature renal arteries contained more proteoglycan signal compared to fetal tissue: 11.8% of the mature signal consisted of proteoglycans, compared to only 1.8% in the fetal selection. Furthermore, there was a dominance of collagens and glycoproteins in both the fetal and mature ECM over other matrisome components (Figure S1C). 

**Figure 2:**
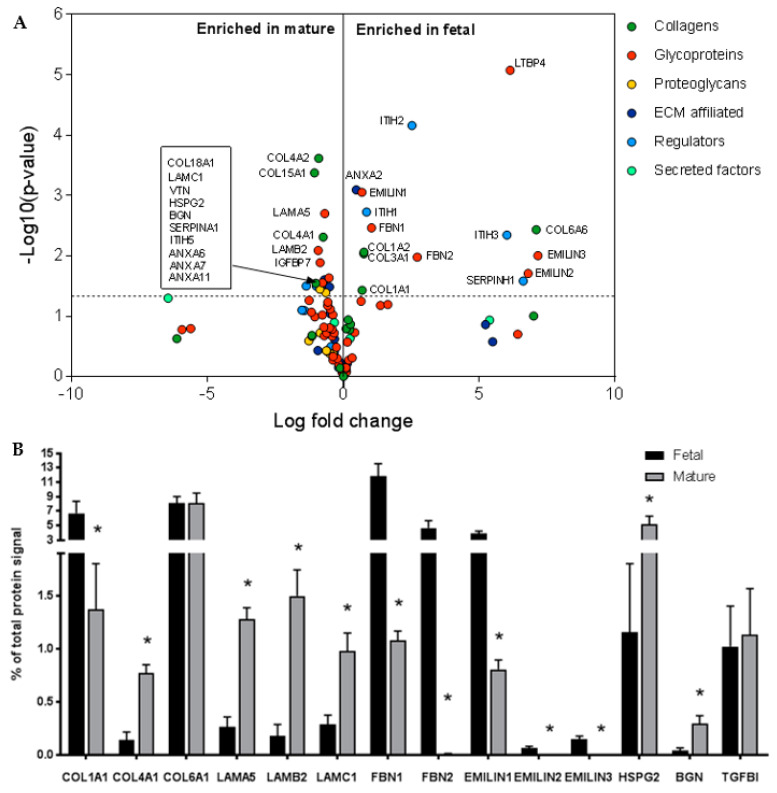
(**A**) Volcano plot represents the protein distribution of fetal and mature extracellular matrix (ECM) proteome fold-changes (x-axis, log fold-change) and significance (y-axis, −log *p*-value). Each circle represents a matrisome protein and each color a specific subset of the matrisome proteins. The horizontal line indicates *p* < 0.05. (**B**) Bar graphs shows examples of ECM proteins identified with LC-MS/MS. Each protein signal is the percentage of the total protein signal. Data are shown as mean ± SEM, *N* = 3, * *p* < 0.05.

Seventy-nine proteins were identified in the fetal and 87 proteins were identified in the mature renal artery ECM pool, from which the majority formed an overlapping core. From this core, the most abundant signal came from collagens and glycoproteins, with many comprising ≥1% of the total signal (Figure S2A). Fold-change of the LFQ intensity showed significant differences between fetal and mature ECM proteins (Figure S2B). Fifteen proteins were significantly enriched in the fetal renal artery ECM compared to the mature ECM (Figure 2A), including collagen type I (Figure 2B). Sixteen proteins were significantly enriched in the mature renal artery ECM compared to the fetal ECM (Figure 2A), including collagen type IV (Figure 2B). Different subtypes of laminins were also significantly more abundant in the mature renal arteries compared to fetal renal arteries (Figure 2B). The proteoglycans HSPG2 and BGN were more abundant in the mature renal arteries as well. The growth factor TGF-β1, which is part of an important signaling pathway involved in both vascular development and vascular quiescence [25], was present in both fetal and mature renal arteries and represented approximately 1% of the total signal. The detection of proteoglycans and growth factors verifies that the used decellularization protocol is gentle enough to preserve low-abundance ECM proteins in the tissue. The full list of all detected fetal renal artery ECM proteins and their relative abundance to total protein composition compared to the mature tissue is available in Table S1. 

### 2.3. Glycoproteins EMILIN1 and FBN1 are Enriched in Fetal Renal Arteries and are Produced by Cells of the Mesenchymal Lineage

**Figure 3:**
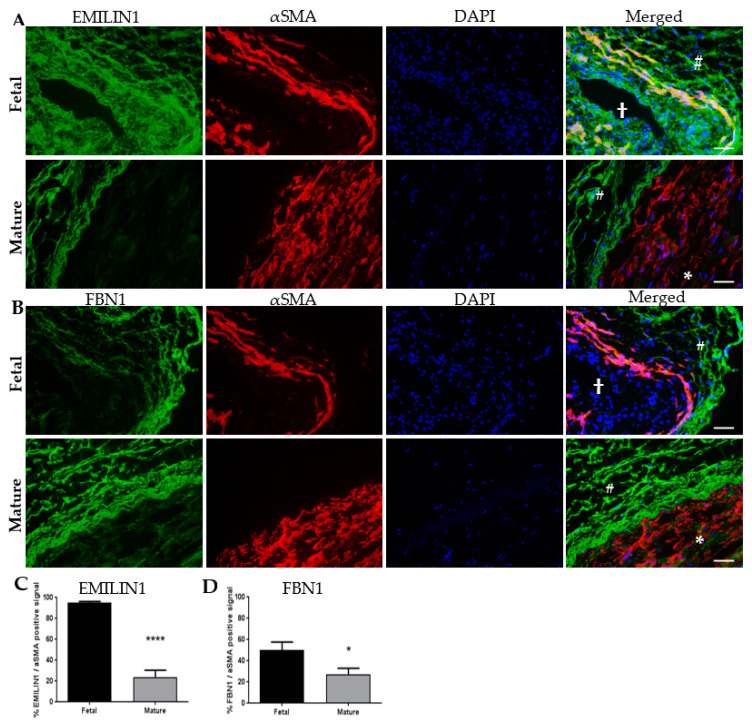
(**A**) Representative images demonstrate the distribution of EMILIN1 co-stained with smooth muscle actin (αSMA) in fetal and mature human renal arteries. Co-localization of αSMA and EMILIN1 in yellow. (**B**) Representative images demonstrate the distribution of FBN1 co-stained with αSMA in fetal and mature human renal arteries. Co-localization of αSMA and FBN1 in yellow. Scale bar represents 50 µm. Open lumen is indicated with a cross, tunica media layer is indicated with an asterix, and the tunica adventitia layer is indicated with a pound sign. (**C**) Quantification of EMILIN1 and αSMA positive signal in percentages in fetal and mature renal arteries. (**D**) Quantification of FBN1 and αSMA positive signal in percentages in fetal and mature renal arteries. Data are shown as mean ± SEM, *N* = 4–5 fluorescent images for EMILIN1 and FBN1 respectively, in fetal samples. *N* = 11–15 fluorescent images for EMILIN1 and FBN1 respectively, in mature samples. * *p* < 0.05, **** *p* < 0.0001 (Student’s *t*-test).

**Figure 4:**
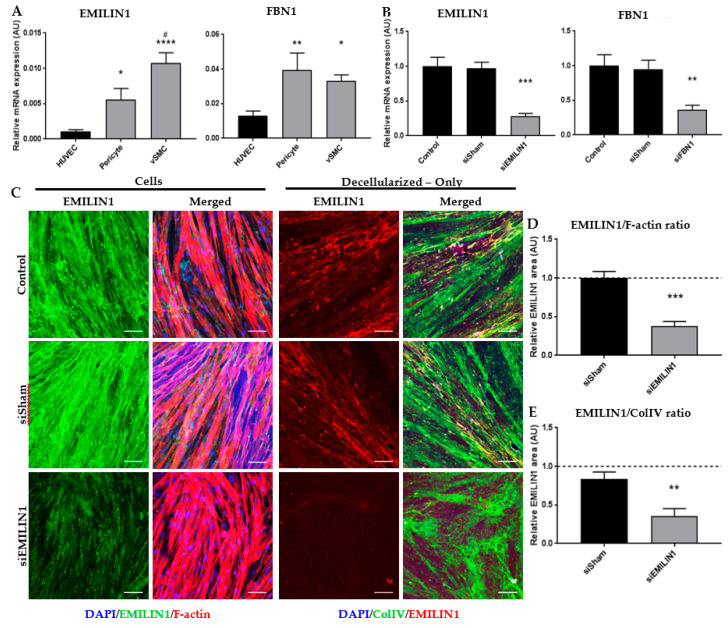
(**A**) qPCR analysis of EMILIN1 and FBN1 in different cell types of the vasculature, human umbilical vein endothelial cells (HUVECs; as endothelium source), pericytes and vascular smooth muscle cells (vSMC; hereafter referred as SMC). Data are shown as mean ± SEM corrected for *beta-actin* (housekeeping gene). *N* ≤ 10, * *p* < 0.05, ** *p* < 0.01, **** *p* < 0.0001 compared to HUVECs, # *p* < 0.05 compared to pericytes (One-way analysis of variance (ANOVA), Tukey’s post hoc test). (**B**) qPCR validation of *EMILIN1* and *FBN1* knockdown in SMC 6 days after siRNA transfection. Data are shown as mean ± SEM, N = 7–9 for *EMILIN1* and *FBN1*, respectively. ** *p* < 0.01, *** *p* < 0.001 compared to siSham (Student’s *t*-test). (**C**) Representative z-stacks of EMILIN1 on SMC cultured for 6 days after siRNA transfection and after decellularization. Scale bar represents 100 µm. (**D**) Quantification of EMILIN1 signal-corrected for the amount of F-actin in siRNA-treated SMC. Data are shown as mean ± SEM, N = 5. (**E**) Quantification of EMILIN1 signal-corrected for the amount of collagen type IV present on SMC-derived ECM coverslips. Data are shown as mean ± SEM, *N* = 7, ** *p* < 0.01, *** *p* < 0.0001 (Student’s *t*-test). Non-treated SMC are set to one (dotted lines).

Elastic components EMILIN1 and FBN1 were significantly more abundant in the fetal renal arteries: 5% and 12% of the total signal were EMILIN1 and FBN1 respectively, compared to only 1% in the mature tissue (Figure 2A,B and Figure S2B). Other EMILIN and FBN members had a higher fold-change in fetal renal arteries compared to mature (Figure S2B) but were less present in the tissue (Figure 2B and Figure S2A). The high protein levels of EMILIN1 and FBN1 in fetal renal arteries hint towards an important role in vascular development and these were therefore selected for follow-up experiments. Candidate proteins EMILIN1 and FBN1 were verified on cross-sections of human fetal and mature renal arteries. Immunohistochemistry indeed showed the presence of the target proteins with dominance in the fetal tissue (Figure 3A–D). EMILIN1 was present in all layers of the fetal renal artery, while in the mature renal artery, it is almost exclusively present in the adventitia. FBN1 was exclusively present in the adventitia of both fetal and mature renal arteries. EMILIN1 and FBN1 co-stained with alpha smooth muscle actin (αSMA) showed more overlap between this mesenchymal marker and the target proteins in the fetal renal artery (Figure 3A–D). Quantification of EMILIN1/FBN1 with αSMA demonstrated that almost 100% and 50% of all αSMA-positive cells were also positive for EMILIN1 and FNB1 respectively, in the fetal vascular tissues. This percentage of overlap declined in mature tissues, demonstrating that the candidate proteins were indeed expressed in fetal tissue by αSMA-positive cells. mRNA expression analysis confirmed a higher expression of *EMILIN1* and *FBN1* in SMCs and pericytes compared to ECs (Figure 4A). This suggests that cells from the mesenchymal lineage produce more EMILIN1 and FBN1 compared to ECs and thereby contribute to the ECM composition of the renal arteries.

### 2.4. ECM Secreted by SMCs Can Be Altered by Depleting Specific ECM Components Using siRNA

Elastic proteins EMILIN1 and FBN1 were abundantly present in fetal renal arteries, suggesting a pivotal role in vascular development. To study the effect of EMILIN1 and FBN1 on the ECM–ECs interaction, a pipeline for a loss of function assay on the ECM level was developed. SMCs in vitro, which produce large amounts of ECM containing EMILIN1 and FBN1, were grown on POMA-FN-modified glass coverslips and were treated with siRNA targeted against EMILIN1 or FBN1. After 6 days, the SMCs were removed, leaving only a coating of SMC-secreted ECM behind that is depleted from either EMILIN1 or FBN1. Anchorage of secreted ECM on coverslips coated with POMA-FN reached an optimum after 6 days of culture, compared with generally used coating methods (Figure S3A–C). QPCR and immunocytochemistry analyses confirmed the knockdown of EMILIN1 or FBN1 in SMCs after 6 days of culturing (Figure 4B–E). After decellularization, the EMILIN1 amount in the anchored ECM was significantly lower compared to sham-treated and untreated controls (Figure 4C,E). Secreted collagen type IV was stained as a reference in the decellularized conditions and showed no difference in the amount of ECM anchored between control and siRNA conditions (Figure S3D). Knockdown of FBN1 in SMCs showed similar results (Figure S4A–C). Thus, using this unique approach, the expression and secretion of EMILIN1 and FBN1 by SMCs can be altered, creating an EMILIN1- or FBN1-depleted ECM layer that can be used in functional assays to study EC behavior. 

### 2.5. Loss of EMILIN1 or FBN1 in the ECM Alters Transcriptome of ECs that Interacted with the Depleted ECM

HUVECs were seeded on EMILIN1- or FBN1-depleted ECM for 24 h, after which the cells were lysed for RNA isolation and processed for RNA-sequencing. In total, 481 and 474 genes were found to be differentially expressed (*p* < 0.05; Figure S5A, Tables S2 and S3) for EMILIN1- and FBN1-depleted ECM respectively, compared to HUVECs seeded on ECM derived from SMC transfected with non-targeting siRNA (control condition). Multiple pathways were identified to be altered in HUVECs cultured on EMILIN1- or FBN1-deficient ECM (Supplemental Tables S4 and S5). A cut-off for the z-score (>2 or <−2) was used to determine the most profound changes. This conical approach revealed the “regulation of actin-based motility by Rho” and “signaling by Rho family GTPases” pathways as a common theme that was decreased in ECs cultured on EMILIN1- or FBN1-deficient ECM, respectively (Figure S5B). 

**Figure 5:**
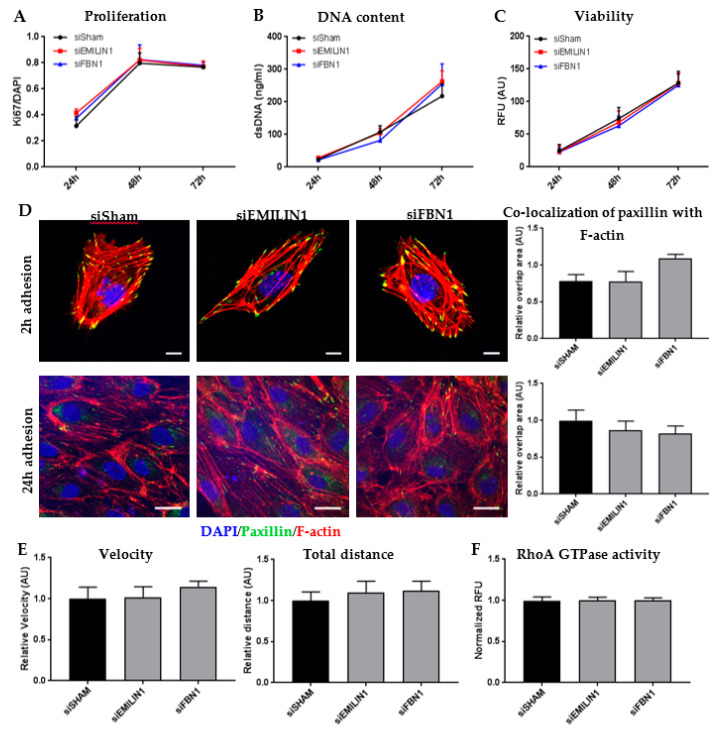
(**A**) Ki67-positive cells/DAPI ratio was used as measure for human umbilical vein endothelial cell (HUVEC) proliferation after 24, 48, and 72 h of culturing on siRNA-treated extracellular matrix (ECM). Shown are mean ± SEM, *N* = 20 fluorescent images per assay, *N* = 3 assays. (**B**) DNA content measurements with the PicoGreen assay was used as measure for HUVEC proliferation after 24, 48, and 72 h of culturing on siRNA-treated ECM. Data are shown as mean ± SEM, *N* = 3. (**C**) HUVEC viability was measured with PrestoBlue after 24, 48, and 72 h of culturing on siRNA-treated ECM. Data are shown as mean ± SEM, *N* = 3. (**D**) Representative z-stacks of HUVECs cultured on siRNA-treated ECM for 2 h (not confluent) to show initial binding to the ECM and 24 h (confluent) to show paxillin and F-actin changes. Scale bar represents 20 µm. Co-localization of paxillin and F-actin were quantified in both assays. Data are shown as mean ± SEM, *N* = 10 fluorescent images per assay, *N* = 4 assays. (**E**) Live cell migration assay results showing the velocity and total distance of HUVECs cultured on siRNA-treated ECM. Data are shown as mean ± SEM, *N* ≈ 10 HUVECs per image, *N* = 10 images per assay, *N* = 6 assays. (**F**) RhoA activation measurements using the G-LISA RhoA GTPase activity assay in HUVECs cultured 24 h on siRNA-treated ECM. Data are shown as mean ± SEM, *N* = 4.

Rho GTPase signaling coordinates cell proliferation by regulating cytoskeleton adaptations [26]. Therefore, in vitro functional assays on cell proliferation and viability were performed to investigate these altered pathways in ECs cultured on EMILIN1- or FBN1-deficient ECM. These assays showed that there was no significant effect of EMILIN1 or FBN1 depletion from the ECM on both EC proliferation and viability (Figure 5A–C). 

Paxillin is a focal adhesion adaptor protein known to be critical for cytoskeleton rearrangements, especially for cell adhesion during migration [27]. According to the pathway analysis, “paxillin signaling” was decreased in HUVECs cultured on EMILIN1-deficient ECM (Table S7). To further evaluate this, an adhesion assay was performed by staining for paxillin in HUVECs after 2 and 24 h of culturing on ECM-altered slides. There was no significant difference in paxillin area in ECs cultured on EMILIN1- or FBN1-depleted ECM (Figure 5D and Figure S6A,B). Furthermore, these ECs did not show any difference on the actin cytoskeleton organization level, although according to the RNA-sequencing data, the pathway “regulation of actin-based motility by Rho” was altered (Figure 5D, Figures S5B and S6A,B). 

Next, a cell migration assay was performed to investigate the motility capabilities of HUVECs cultured on EMILIN1- or FBN1-deficient ECM. Overnight migration and tracking of individual cells could not identify a significant effect of EMILIN1 and FBN1 depletion on the migratory capabilities of HUVECs (Figure 5E and Figure S6C). Also, a RhoA GTPase activation assay showed no differences between HUVECs cultured on EMILIN- or FBN1-deficient ECM compared to control ECM (Figure 5F). 

These findings indicate that depletion of EMILIN1 or FBN1 from the ECM only has a clear effect on the transcriptome response of ECs in the current setting, but does not influence their functional behavior with respect to adhesion, migration and proliferation.

## 3. Discussion

In this study, we characterized the ECM proteins present in fetal and mature renal arteries and focused on the impact of two elastic proteins on ECs: EMILIN1 and FBN1. Our major findings are: (1) Enriching the ECM in human vascular tissue by decellularization aids in the detection of ECM proteins using LC-MS/MS. (2) The ECM protein expression differs between fetal (growth-induced) and mature (quiescent) vascular tissue. (3) Next to 13 other proteins, elastic ECM proteins EMILIN1 and FBN1 are significantly enriched in fetal renal arteries and are mainly produced by cells of mesenchymal origin. (4) SMCs-secreted ECM can be altered and tightly anchored to POMA-FN coverslips, creating a platform to study EC–matrix interactions. (5) EMILIN1- and FBN1-deficient ECM provoked alterations in HUVECs via multiple pathways, especially Rho GTPase-controlled pathways. (6) However, EMILIN1 and FBN1 ECM components only play a minor role in EC behavior. 

Our proteomic approach to unravel the differences between the fetal and mature vascular ECM has the potential to find interesting proteins that can influence EC behavior. Decellularization assured enrichment of ECM proteins before proteomic analysis. Not all cellular components were removed, but it guaranteed an enriched ECM fraction of 38% and 35% in fetal and mature conditions, respectively. Protein lysates of whole tissue contain only 5% ECM proteins of the total amount of proteins, confirming the necessity to enrich for ECM proteins. Detergents for decellularization can be very stringent, however fragile components of the ECM (proteoglycans, secreted factors) were still detectable. 

Many ECM proteins were identified by cross-referencing with the Human Matrisome Project [3,10] and differences in protein abundance were observed between active (fetal) and quiescent (mature) vascular ECM. Overall, elastic proteins, such as members from the EMILIN/multimerin and fibrillin families, were more abundant in the fetal ECM compared to the mature ECM, while the reverse is observed for the laminin family. Although it was already known that the mature basement membrane is rich in laminin [5], the distribution of multiple isoforms of laminin in the active and quiescent vascular ECM is now demonstrated by our dataset. Furthermore, this observation is in line with the hypothesis that a quiescent, more mature phenotype arises in ECs when cultured on a laminin coating compared to a collagen coating [6]. 

Multiple members of the EMILIN/multimerin and fibrillin families were significantly more abundant in the fetal renal artery ECM compared to the mature ECM, namely: EMILIN-1, -2 and -3 and fibrillin-1 and -2. Fibrillin-2 is needed for the initial assembly of the aortic matrix during development and overlaps with fibrillin-1 expression [28]. These findings indicates an important developmental role for fibrillin-2. More proteins in our vascular ECM catalogue could represent interesting targets for further research, especially ECM interaction proteins such as members from the MAGP and LTBP families. Mutations in Microfibril-Associated Protein 5 (MFAP5), a member of the MGAP family, are linked to aortic aneurysms and dissections [29]. In concordance, double knockout of both MFAP-2 and -5 in mice results in aortic dilation [30]. These studies indicate that MFAPs may contribute to maintaining large vessel integrity. Latent Transforming Growth Factor Beta Binding Proteins (LTBPs) attach to both fibrillins and latency-associated protein of TGF-β (LAP), thereby forming a TGF-β reservoir within the ECM [31,32]. LTBP (1–4)-null mice exhibit severe phenotypes, including defects in bone, lung, and cardiovascular development [32]. These are all examples of promising ECM proteins detected in this study that are known to be important during vascular development. These may be interesting candidates to improve the bioactivity of scaffolds in vascular grafts. Moreover, we also detected the majority of these elastic ECM proteins in a proteome screen of fetal versus mature renal tissue [24]. This suggests that elastic components of the ECM are not only important in vascular development, but also in renal development, and presumably in general embryonic development.

Due to their abundant presence in our vascular ECM catalogue compared to other family members, elastic proteins EMILIN1 and FBN1 were selected in this study to further elucidate their impact on EC fate. The role of EMILIN1 is extensively studied in EMILIN1-deficient mice, which display aortic valve malformations and hypertension [21,33]. EMILIN1 plays a pivotal role in mice blood vessel development and elastogenesis [34,35]. Most of the diseases accompanied by EMILIN1 deficiency are driven by TGF-β. In the vasculature, EMILIN1 functions as an antagonist in the processing steps of TGF-β and thereby regulates vascular tone and blood pressure [36,37,38]. EMILIN1 has multiple domains which exhibit different functions in the vasculature. The EMI domain is linked to hypertension and TGF-β processing [36], while adhesive functions of EMILIN1 are related to its gC1q domain. The gCq1 domain regulates cell migration and proliferation via a specific interaction with α4β1 integrin [18,20,39]. Integrin α9β1 shows a similar interaction with the gC1q domain and plays a role in lymphatic EC migration and proliferation [18,19].

Similar to EMILIN1, FBN1 can mediate the adhesion and migration of several cell types, including ECs [40]. Endothelium dysfunction is an important contributor to Marfan syndrome, caused by mutations in the FBN1 gene. A heterozygous FBN1 mutation in mice accelerates vascular aging and eventually leads to aortic manifestations, resembling those of Marfan syndrome [22]. 

The data presented in this study focus on the role of EMILIN1 and FBN1 on ECs adhesion, migration, and proliferation. Although RNA-sequencing pointed towards a role of EMILIN1 and FBN1 in Rho-mediated cytoskeleton rearrangements, which is required for migration and proliferation, functional assays revealed that ECs are not altered in their capacity to divide and migrate when either EMILIN1 or FBN1 is depleted from the ECM. This is in contrast with the existing literature on the effect of EMILIN1 and FBN1 on proliferation and migration [24]. This discrepancy may be attributed to differences in study design and are potential limitations in this study. We cultured our cells on a complex ECM network depleted from either EMILIN1 or FBN1 from the beginning, rather than using protein fragments of EMILIN1 or FBN1 as coating, which is a strategy often used in other EMILIN1 or FBN1 in vitro studies [17,19,41,42]. A possible combination of both EMILIN1- and FBN1-deficient ECM may demonstrate a synergistic effect. Furthermore, although both candidate proteins are prominently present in the fetal ECM, they are potentially less effective compared to family members or other candidate proteins such as the LTBP and MFAP family. Further studies should explore the (synergistic) effects of other interesting proteins in vascular development. The environment created by our protocol resembles the complex ECM in vivo more closely, however this approach might not be ideal to pick up small behavioral changes. Conversely, our recent study shows that renal cells cultured using the exact same 2D ECM coating approach do respond to depletion of EMILIN1 by reducing their adhesive strength and subsequently adopting a more migratory phenotype [24]. It is therefore possible that the lack of response as observed in this study, is cell type-specific, and that ECs are perhaps less reliant on both candidate proteins compared to renal cells for adapting their cell behavior to ECM [24]. It is possible that the HUVECs used in our study express an integrin profile that is less sensitive to changes in EMILIN1 or FBN1 level. HUVECs have been used as the golden standard in vascular research since the late 1970s [43] and are considered to be a robust cell model with a high proliferation rate. Using a different and more sensitive type of EC instead, for example human microvascular endothelial cells (HMVECs) or endothelial colony-forming cells (ECFCs), might prove to be more suitable to test EC behavior. For example, in line with this hypothesis, it has been shown that EMILIN1 can regulate the proliferation of HMVECs and acts as a guiding molecule during their migration by interacting with α9 integrins actively expressed by these cells [19]. 

In conclusion, the addition of EMILIN1 or FBN1 to scaffolds for cell seeding of HUVECs most likely will not have an effect on their adhesive, migratory and proliferative behavior. Nevertheless, EMILIN1 or FBN1 might still be an interesting candidate for implementing in scaffolds containing other (vascular) cell types, as studies have clearly identified these elastic proteins as regulators of cell adhesion and migration. The presented ECM protein catalogue could help in identifying valuable target proteins for the next phase of vascular tissue engineering.

## 4. Materials and Methods

### 4.1. Human Tissue

Healthy, age- and sex-matched human fetal and mature renal artery tissue was used for ECM analysis by liquid chromatography tandem mass spectrometry (LC-MS/MS). Residual fetal renal arteries were obtained from the department of Molecular Cell Biology at the Leiden UMC. Residual mature renal arteries were obtained from the Erasmus MC biobank for rest material used for diagnostics. All samples were fresh-frozen and stored at −80 °C prior to use. Sampling and handling and use of tissues were approved by the medical ethical committees of the Erasmus MC and Leiden UMC (project code 108017 (26-4-2019)). 

### 4.2. Sample Preparation

To enrich the ECM protein content, renal arteries were decellularized by several detergents. First, 1% SDS was used to decellularize the tissue for 12 h. Next, 1% Triton X-100 was used for 1 h. The samples were washed for 2 h with PBS to remove the detergents. All steps were performed at room temperature and under constant rotation. Frozen sections of decellularized and non-decellularized tissue were used for hematoxylin and eosin (HE) staining to confirm complete decellularization of the renal arteries while preserving the ECM architecture.

Decellularized renal arteries were homogenized in lysis buffer (10 mM Tris (pH 7.4), 100 mM NaCl, 0.1% SDS, 0.5% sodium deoxycholate, 1% Triton X-100, 1 mM EGTA, 1 mM EDTA, 10% Glycerol, 1 mM NaF, 1 mM sodium orthovanadate, and a protease inhibitor cocktail (Roche, Mannheim, Germany)) using an Ultra-Turrax (IKA, Staufen, Germany). Lysates were placed on ice for 30 min and cell debris was pelleted via centrifugation (10 min, 1000× *g*, 4 °C). A pool of either 2 mature or 3 fetal renal arteries was considered as 1 sample for sufficient protein yield (Table S6). Tissue lysates were separated by SDS-PAGE on an equally loaded pre-cast 4%–12% linear gradient gel (NuPAGE Bis-Tris Mini gels, Life Technologies, Bleiswijk, The Netherlands) and visualized with Coomassie Blue (Figure S1A). LC-MS/MS analysis was performed in triplicate. 

### 4.3. LC-MS/MS Analysis

SDS-PAGE-separated samples were prepared according previously used protocols for LC-MS/MS analysis by the Proteomics Centre of the Erasmus MC [24]. In short, proteins lanes were cut out of the gel, reduced with dithiothreitol, alkylated with iodoacetamide, and digested with trypsin, as described previously [44]. Supernatants were stored in glass vials at −20 °C until further measurements. An 1100 series capillary LC system (Agilent Technologies, Amstelveen, The Netherlands) was used for nanoflow LC-MS/MS coupled to an LTQ-Orbitrap XL mass spectrometer (Thermo, Landsmeer, The Netherlands) operating in positive mode and equipped with a nanospray source, as previously described [45]. Samples were trapped on a 1.5 cm × 100 µm in-house packed ReproSil C18 reversed phase column (Dr Maisch GmbH, Ammerbuch-Entringen, Germany) at a flow rate of 8 µL/min. Sequentially, samples were separated on a 15 cm × 50 µm in-house packed ReproSil C18 reversed phase column (Dr Maisch GmbH) by adding a linear gradient from 0%–80% solvent B in solvent A, where A consisted of 0.1 % formic acid and B of 80% (*v*/*v*) acetonitrile and 0.1 % formic acid. Flow rate was set at 200 nL/min, and elution took place over 70 min. The eluent was sprayed by a nanospray device directly into the ESI source of the LTQ ion trap mass spectrometer. Mass spectra were acquired in continuum mode, and peptide fragmentation was performed in data-dependent mode. MS/MS spectra were extracted out of raw data files and were analyzed by using MaxQuant software, as previously described [46]. 

### 4.4. MS Data Analysis

First, analysis was done with proteins which were present in at least 2 fetal or 2 mature sample groups. Second, data from the Human Matrisome project [3,10] was used to filter ECM core and associated proteins from the generated datasets. Label-free quantification (LFQ) intensity was used to further specify the abundance of ECM components in the fetal and mature renal artery. Differences in the abundance of ECM components between fetal and mature tissue was showed as a percentage of the total ECM protein signal.

### 4.5. Immunohistochemistry

Protein validation was performed by immunohistochemistry on 7 µm thick frozen sections of non-decellularized tissue. Frozen sections were used for a standard hematoxylin and eosin staining and for a protein-specific staining using fluorescent secondary antibodies. Acetone-fixed sections were blocked with 1% BSA in PBS for 1 h. Polyclonal primary antibodies against EMILIN1 and FBN1 were diluted in 1% BSA/PBS (1:100 and 1:200, respectively; both Sigma Aldrich, Zwijndrecht, The Netherlands) and incubated for 1 h at room temperature. Sections were washed 3 times in PBS/T solution and incubated with Alexa Fluor 488 donkey anti-goat IgG (1:100; Life Technologies) and αSMA-Cy3 (1:500; Sigma) diluted in 1% BSA/PBS for 1 h at room temperature. After 3 times washing with PBS/T solution, sections were incubated with DAPI for 5 min, washed with PBS, and mounted. Stained sections were imaged using fluorescent microscopy (Olympus IX53, Olympus Leiderdorp, The Netherlands) and ImageJ (version 1.47) to analyze EMILIN1, FBN1, and αSMA positive area. 

### 4.6. Cell Culture

Human umbilical vein endothelial cells (HUVECs; Lonza, Basel, Switzerland) and HUVECs transfected with lentiviral green fluorescent protein (GFP) were cultured on gelatin-coated plates in endothelial growth medium (EBM-2 basal medium supplemented with EGM-2 bullit kit; Lonza) in 5% CO_2_ at 37 °C. Human aorta smooth muscle cells (SMCs; Lonza) were cultured on gelatin-coated plates in smooth muscle growth medium (SMBM basal medium supplemented with SMGM bullet kit; Lonza) in 5% CO_2_ at 37 °C. Experiments were performed with cells between passage 3 and 6. Knockdown of the ECM components FBN1 and EMILIN1 were performed by their specific ON-TARGETplus SMARTpool siRNAs (Dharmacon, Horizon Discovery, Cambridge, UK) and DharmaFECT-1 (Dharmacon), with a final concentration of 200 nM. ON-TARGETplus Non-targeting pool (siSham; Dharmacon) was used as a negative control (Table S7).

### 4.7. POMA Slides for Tight Anchoring of ECM

To anchor secreted ECM proteins, coverslips were treated following the protocol as described by Labit et al. [47]. Briefly, contaminants on coverslips (ø18 mm; VWR, Amsterdam, The Netherlands) were removed by serially washing with acetone, methanol/water, and chloroform in combination with sonication. Coverslips were oxidized using piranha solution consisting of sulfuric acid (99.999%; Sigma) and hydrogen peroxide (35% wt. in H2O, Merck Millipore, Amsterdam, The Netherlands) in a ratio of 7:3, followed by salinization with a 2% (3-Aminopropyl)triethoxysilane (APTES, 99%; Sigma) solution in 95% ethanol. A poly(maleic anhydride-alt-1-octadecene) (POMA; Sigma) layer was applied by spin-coating at 4000 rpm for 30 s, using a 0.16% solution of POMA in tetrahydrofuran (Sigma). Polymerized coverslips were sterilized with UV-light and used immediately or stored in the dark at room temperature. Prior to cell culture, POMA-treated coverslips were coated with 50 µg/mL fibronectin (Roche) in PBS for 1 h at 37 °C, creating POMA-FN coverslips. 

SMCs were harvested and seeded onto POMA-FN at 150,000 cells per well. After transfection with siRNA, cells were cultured for six days in order to produce sufficient ECM. Decellularization was achieved by mild agitation in combination with warm 20 mM ammonium hydroxide. Light microscopy was used to confirm whether decellularization was complete. Coverslips were incubated with DNase (Qiagen, Venlo, The Netherlands) for 15 min at room temperature to remove DNA traces. Finally, decellularized ECM layers were washed in ultrapure water and PBS to remove cellular residues.

### 4.8. Immunocytochemistry

Prior to fixation, both control and decellularized ECM layers were washed in PBS twice. Samples were fixated with 4% paraformaldehyde (PFA), permeabilized with 0.1% Triton X-100, and blocked with 1% BSA in PBS. Samples were incubated for 1 h with collagen IV antibody (1:50, Millipore, Amsterdam, The Netherlands), EMILIN1 antibody (1:100; Sigma), or FBN1 antibody (1:200; Sigma). Samples were incubated with Alexa Fluor 488 donkey anti-goat IgG (1:100, Life Technologies) or Alexa Fluor 488 goat anti-rabbit IgG (1:100, Life Technologies), and Alexa Fluor 596 donkey anti-goat IgG (1:100; Life Technologies) or rhodamine-phalloidin (1:40; Life Technologies) in the dark for 1 h. Nuclei were counterstained with DAPI (1:5000) for 15 min. Washing with 0.05% Tween in PBS occurred after every antibody incubation. Samples were mounted on object slides using Mowiol. Imaging was performed using a Leica SP8X confocal microscope (Leica Microsystems, Amsterdam, The Netherlands) and LAS X software. Z-stacks were made to capture all fluorescent signal (5 a 6 Z-stacks/coverslip). Projections of the z-stack images were analyzed with ImageJ to calculate the area of EMILIN1, FBN1, collagen IV, and F-actin. 

### 4.9. Quantitative PCR

RNA was isolated at indicated time points using the ISOLATE II RNA kit (Bioline, GC biotech, Waddinxveen, The Netherlands) and cDNA was made using the SensiFAST cDNA synthesis kit (Bioline) according to the manufacturer’s protocol. qPCR was performed using FastStart Universal SYBR Green Master (Roche) according the following qPCR program: 8.5′ 95 °C, 38 cycles (15” 95 °C; 45” 60 °C) 1′ 95 °C, 1′ 65 °C, 62 cycles (10” 65 °C + 0.5 °C). Expression levels are relative to housekeeping gene beta actin. Primer sequences are listed in Table S8.

### 4.10. Endothelial Cell Assays

HUVECs were reseeded with 35,000 cells per coverslips or indicated otherwise and cultured for various times on SMC-derived non-targeted, EMILIN1- or FBN1-deficient ECM coverslips in a 12-well plate. ECs were cultured in full EGM-2 medium or indicated otherwise, in 5% CO_2_ at 37 °C.

#### 4.10.1. RNA Sequencing

RNA was isolated from HUVECs using the ISOLATE II RNA kit after 24 h culturing. RNA sequencing was done as previously described [48]. Briefly, sequencing libraries were made from poly-adenylated RNA using the Rapid Directional RNA-Seq Kit (NEXTflex, Perkin Elmer, Austin, TX, USA) and sequenced on Illumina NextSeq500 to produce single-end 75-base long reads (Utrecht Sequencing Facility). Reads were aligned to the human reference genome GRCh37 using STAR version2.4.2a. Read groups were added to the BAM files with Picard’s AddOrReplaceReadGroups (v1.98). The BAM files were sorted with Sambamba v0.4.5, and transcript abundances were quantified with HTSeq-count version 0.6.1p117 using the union mode. Subsequently, reads per kilobase of transcript per million reads sequenced were calculated with edgeR’s rpkm() function. RNA-sequencing results were analyzed using Qiagen’s Ingenuity Pathway Analysis (IPA). IPA was used to identify pathways that were altered by differentially expressed genes. *p*-values were calculated by IPA based on a right-tailed Fisher’s Exact Test.

#### 4.10.2. Proliferation Assay

HUVECs were fixed with 4% PFA after 24, 48, and 72 h of culturing. Proliferation was assessed by staining with a Ki67 antibody (1:200; ThermoScientific, Landsmeer, The Netherlands) using the above-described immunocytochemistry protocol. Coverslips were imaged using a Leica DM 5500B microscope and images were quantified for Ki67^+^ and DAPI^+^ cells using ImageJ.

#### 4.10.3. PicoGreen Assay

HUVECs were isolated from the ECM coverslips after 24, 48, and 72 h culturing for dsDNA measurements using PicoGreen, according to the manufacturer’s protocol (ThermoFisher, Landsmeer, The Netherlands).

#### 4.10.4. PrestoBlue Assay

Viability was measured using PrestoBlue Cell Viability Reagent (ThermoScientific) in the medium of HUVECs cultured on siRNA-treated ECM for 24, 48, and 72 h, according to the manufacturer’s protocol.

#### 4.10.5. Adhesion Assay

After 2 and 24 h, HUVECs were fixed with 4% PFA. Adhesion was assessed by staining with a paxillin antibody (1:100; Abcam, Cambridge, UK) using the above-described immunocytochemistry protocol. Projection of the z-stack images were analyzed using ImageJ to calculate the area of paxillin, F-actin (phalloidin), and the co-localization of this focal adhesion protein with the cytoskeleton of the cells.

#### 4.10.6. Migration Assay

HUVECs-GFP (to visualize the cells) were seeded with 25,000 cells per coverslip and live-imaged overnight using a Leica SP8X confocal at 37 °C and humid atmosphere containing 5% CO_2_. Single cell migration was imaged every 15 min at 10 positions in each condition, ending after 24 h of culturing. Obtained videos were analyzed using ImageJ Manual Tracking and Chemotaxis Tool.

#### 4.10.7. RhoA GTPase Activity Assay

HUVECs were seeded with 200,000 cells per coverslip and starved overnight in bare EBM-2 medium and stimulated to activate RhoA for 5 min in full EGM-2 medium 24 h after seeding. Cells were lysed for GTPase activity using the G-Lisa RhoA Activation Assay Colorimetric Kit (Cytoskeleton, Denver, CO, USA) according to the manufacturer’s protocol. 

### 4.11. Statistical Analysis

All results were analyzed and presented using GraphPad Prism 6. All statistical comparisons were made by performing a Student’s T-test or a one-way analysis of variance (ANOVA) followed by a Tukey’s multiple comparison test. Error bars were visualized as standard error of the mean. *p*-values < 0.05 were considered statistically significant.

## Figures and Tables

**Table 1:** Number of genes detected in the fetal and mature renal artery group, divided into matrisome (ECM) proteins and non-matrisome proteins. Within the group of matrisome proteins, different compartments are recognized as defined by the matrisome project: http://web.mit.edu/hyneslab/matrisome/. Core matrisome proteins comprise the building blocks of the ECM and include collagens, glycoproteins, and proteoglycans. In addition, matrisome-associated proteins are comprised of ECM binding proteins, which include ECM-affiliated proteins, ECM regulators, and secreted factors. Proteins were present in at least 2 out of 3 pools in both fetal and mature pooled samples.

Proteins Detected	Fetal Renal Artery	Mature Renal Artery
Total number of proteins detected	206	246
Matrisome proteins	79 (38.3% of total)	87 (35.4% of total)
Non-matrisome proteins	127 (61.7% of total)	159 (64.6% of total)
Matrisome core proteins	58 (73.4% of matrisome)	63 (73.3% of matrisome)
Glycoproteins	38 (65.6% of core)	39 (61.9% of core)
Collagens	14 (24.1% of core)	15 (23.8% of core)
Proteoglycans	6 (10.3% of core)	9 (14.3% of core)
Matrisome-associated proteins	21 (26.6% of matrisome)	23 (26.7% of matrisome)
ECM-affiliated	9 (42.9% of associated)	9 (39.1% of associated)
ECM regulators	7 (33.3% of associated)	10 (43.5% of associated)
Secreted factors	5 (23.8% of associated)	4 (17.4% of associated)

## Supplementary Materials

Supplementary materials can be found at https://www.mdpi.com/1422-0067/21/11/3905/s1.

## Abbreviations

APTES	(3-Aminopropyl)triethoxysilane
αSMA	Alpha-smooth muscle actin
EC	Endothelial cell
ECM	Extracellular matrix
EGM-2	Endothelial growth medium-2
EMILIN1	Elastin microfibril interfacer 1
FBN1	Fibrillin-1
FN	Fibronectin
HUVEC	Human umbilical vein endothelial cell
LC-MS/MS	Liquid chromatography tandem mass spectrometry
POMA	poly(maleic anhydride-alt-1-octadecene)
POMA-FN	poly(maleic anhydride-alt-1-octadecene) with fibronectin
SMC	Smooth muscle cell
TGF-β	Transforming growth factor beta
