# Supplementary material for: Extracellular Matrix Analysis of Human Renal Arteries in Both Quiescent and Active Vascular State
Source: Int J Mol Sci. 2020 May 30;21(11):3905. doi: 10.3390/ijms21113905 (PMC7313045; doi:10.3390/ijms21113905)
Supplement: Supplementary file 1 [file ijms-21-03905-s001.pdf]

Supplemental figures

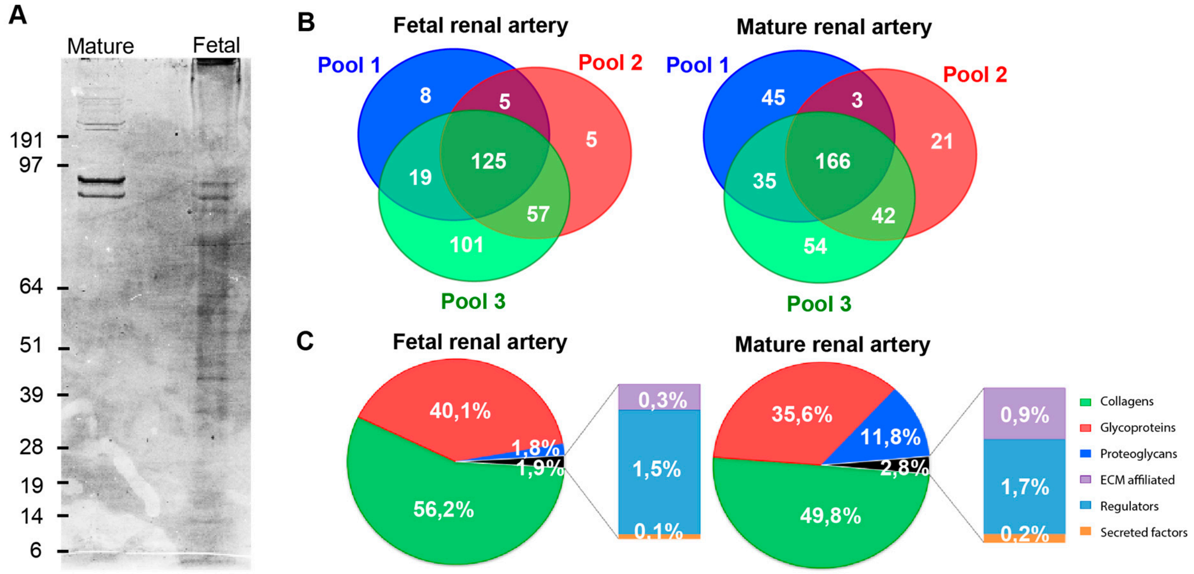

Supplemental Figure 1. (**A**) Extracellular matrix (ECM) lysates from human fetal and mature renal arteries were separated by SDS-PAGE and stained with Coomassie Blue prior to LC-MS/MS analysis. Shown is a representative blot of 3 experiments containing pooled renal arteries samples (fetal or mature). (**B**) Venn diagrams showing the total amount and overlap of proteins identified in the three pools of either fetal and mature samples by LC-MS/MS. Proteins identified in at least 2 pools samples were used for further analysis. (**C**) Pie charts showing the distribution of the six matrisome classes in percentages of the LFQ intensity compared to LFQ intensity of the total matrisome present in fetal and mature renal arteries.

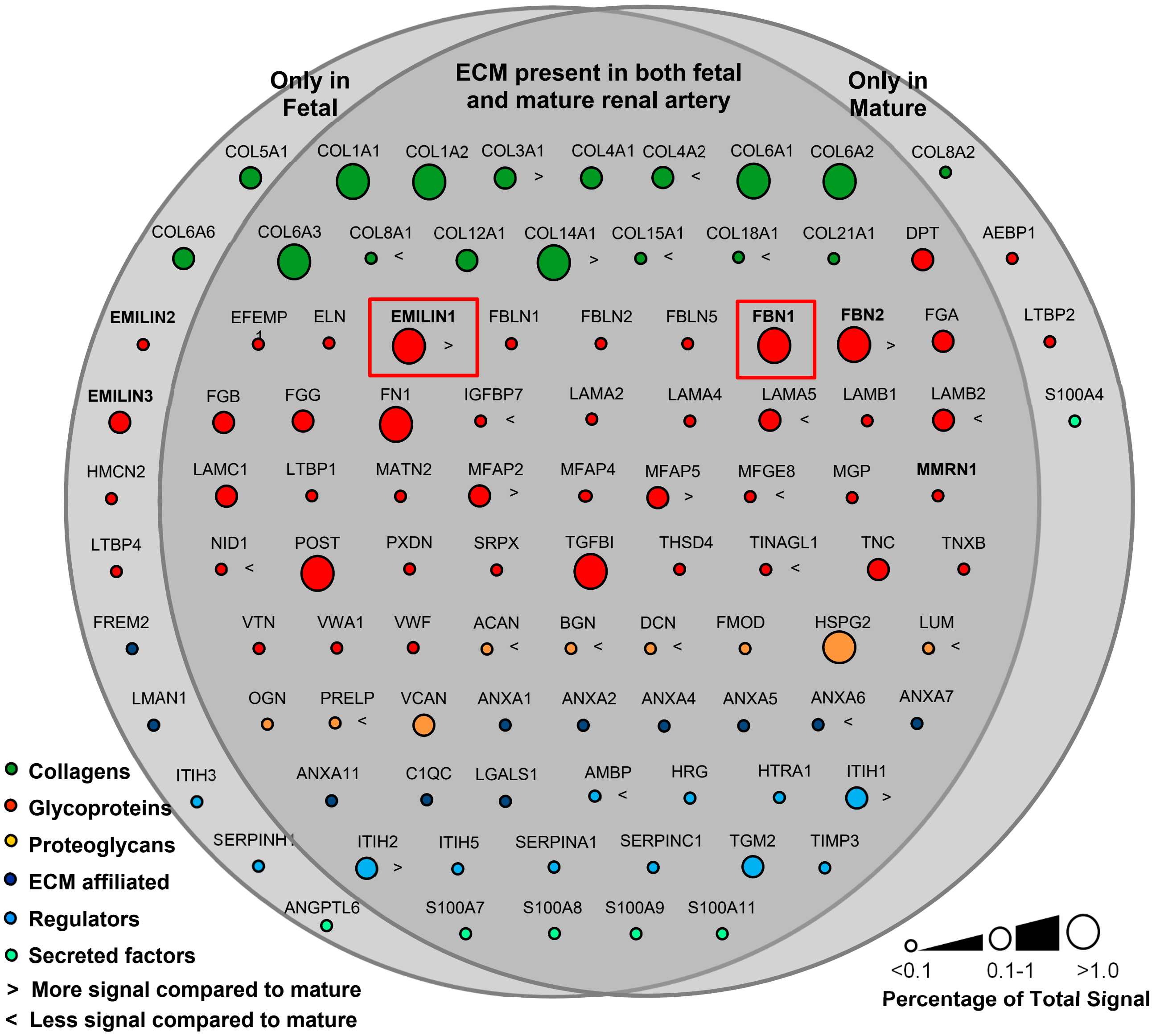

Supplemental Figure 2. (**A**) Euler-diagram visualizing the overlap and differences between the human fetal and mature renal artery proteome. Each node represents an ECM protein labeled with the gene name. Node size represents protein abundance in percentages of the total protein signal. Proteins of interest EMILIN1 and FBN1 are highlighted in bold and with a red box, their family members highlighted in bold. Shown are the means of all pooled samples (N=3).

B

Protein ID	Log FC	P-value
**Glycoproteins**		
**EMILIN3**	4,15	0,010
**EMILIN2**	3,77	0,020
**FBN2**	3,57	0,011
MFAP2	3,15	0,064
LTBP4	3,15	0,000
HMCN2	2,36	0,199
MFAP5	2,19	0,066
PXDN	1,93	0,057
THSD4	1,18	0,186
**FBN1**	1,03	0,003
**MMRN1**	1,01	0,490
ELN	0,91	0,503
**EMILIN1**	0,69	0,001
POSTN	0,17	0,269
VWF	0,12	0,535
FGA	0,02	0,631
TGFBI	-0,04	0,858
FBLN5	-0,07	0,782
TNXB	-0,14	0,533
FGG	-0,31	0,190
FGB	-0,36	0,165
FN1	-0,46	0,077
LAMC1	-0,57	0,023
TNC	-0,63	0,215
LAMA2	-0,68	0,467
MATN2	-0,71	0,834
LAMA5	-0,77	0,002
LAMA4	-0,83	0,746
LAMB1	-0,91	0,715
LAMB2	-1,12	0,008
LTBP1	-1,17	0,327
SRPX	-1,20	0,211
EFEMP1	-1,31	0,059
FBLN2	-1,39	0,095
FBLN1	-1,42	0,156
VWA1	-1,44	0,095
VTN	-1,68	0,028
MFAP4	-1,76	0,103
AEBP1	-1,85	0,161
DPT	-1,86	0,191
NID1	-1,96	0,067
IGFBP7	-1,97	0,013
LTBP2	-2,06	0,167
MGP	-2,47	0,152
MFGE8	-2,91	0,055
TINAGL1	-3,19	0,086

Protein ID	Log FC	P-value
**Collagens**		
COL6A6	4,09	0,004
COL5A1	3,91	0,099
COL3A1	1,94	0,009
COL1A2	0,78	0,009
COL1A1	0,69	0,037
COL14A1	0,28	0,136
COL12A1	0,21	0,168
COL6A3	0,19	0,115
COL6A2	0,10	0,162
COL6A1	0,01	0,995
COL21A1	-0,09	0,724
COL15A1	-1,97	0,000
COL4A1	-2,01	0,005
COL8A2	-2,14	0,237
COL4A2	-2,23	0,000
COL8A1	-2,78	0,208
COL18A1	-3,25	0,028
**Proteoglycans**		
OGN	-1,28	0,417
ACAN	-1,69	0,188
BGN	-1,95	0,036
DCN	-2,05	0,257
PRELP	-2,83	0,216
**Secreted Factors**		
ANGPTL6	1,71	0,116
S100A7	0,90	0,959
S100A8	0,25	0,233
S100A11	-1,07	0,128
S100A9	-1,30	0,194
S100A4	-3,38	0,050

Protein ID	Log FC	P-value
**ECM Affliated**		
FREM2	1,70	0,267
LMAN1	1,60	0,138
ANXA2	0,48	0,001
ANXA5	-0,12	0,599
ANXA1	-0,49	0,091
LGALS1	-0,97	0,614
C1QC	-1,20	0,371
ANXA4	-1,22	0,240
ANXA11	-1,48	0,038
ANXA6	-1,71	0,033
ANXA7	-2,18	0,025
LUM	-0,24	0,417
VCAN	-0,34	0,374
HSPG2	-0,83	0,041
FMOD	-0,91	0,379
**ECM Regulators**		
SERPINH1	3,59	0,026
ITIH2	3,26	0,000
ITIH3	3,01	0,005
ITIH1	0,88	0,002
HRG	-0,22	0,391
TIMP3	-0,33	0,320
TGM2	-0,60	0,088
SERPINC1	-0,90	0,711
ITIH5	-2,49	0,032
SERPINA1	-2,53	0,032
HTRA1	-2,84	0,081
AMBP	-3,26	0,080

Supplemental Figure 2 Continued. (**B**) Heat map of fetal vs mature fold change. Fold changes were calculated by the LFQ intensity percentages in fetal compared to mature renal artery. Both core and associated matrisome proteins are included in the heat map. Proteins of interest EMILIN1 and FBN1 are highlighted in bold and with a red box, their family members highlighted in bold. Sample size N=3. Student t-test was used for statistical analysis.

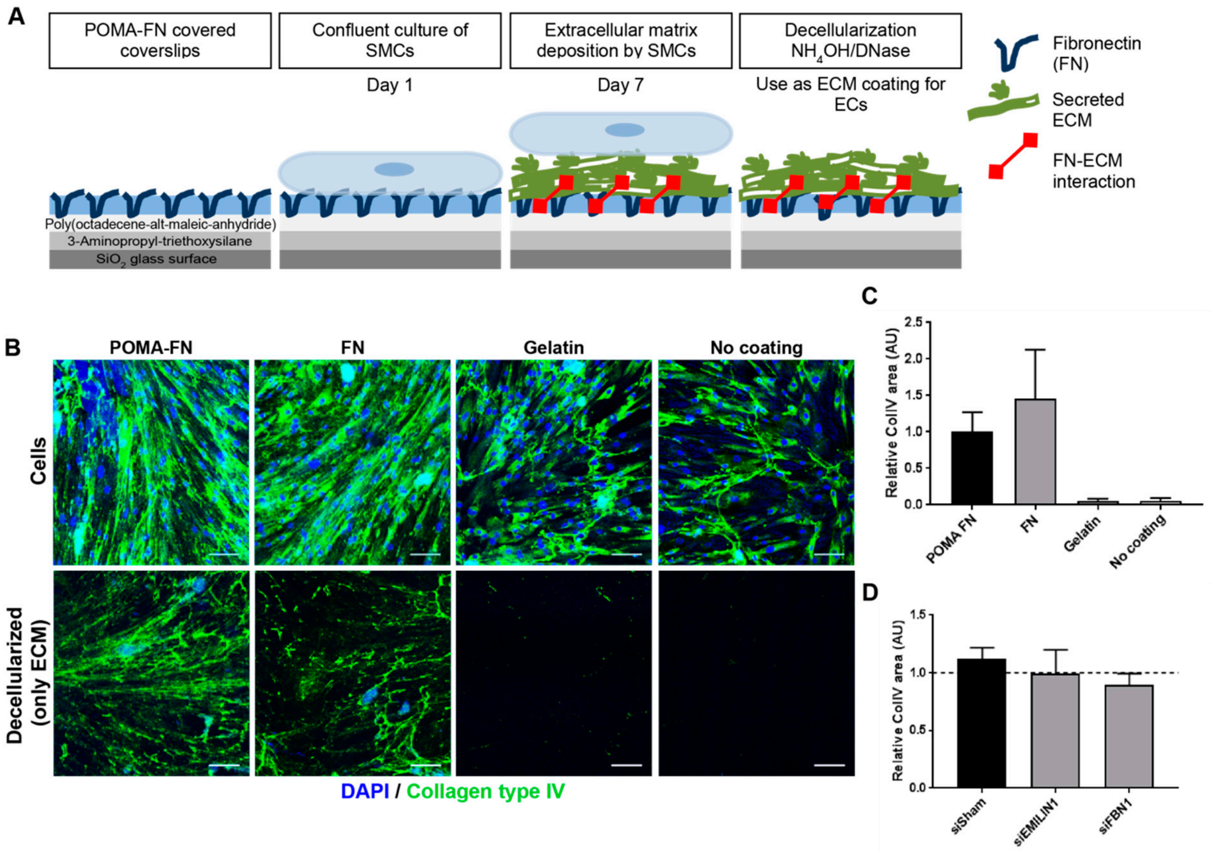

Supplemental Figure 3. (**A**) Timeline for the production of smooth muscle cell (SMC) derived extracellular matrix (ECM) on poly(octadecene-alt-maleic) anhydride-fibronectin (POMA-FN) treated coverslips. First, coverslips were oxidized using piranha solution to link silane to the aminosilane (APTES) group. POMA binds to APTES that can covalently bond fibronectin (FN). Over time, SMCs deposit ECM, which covalently bonds to FN, anchoring the secreted ECM. Ammoniumhydroxide (NH_4OH) and DNase remove all traces of the SMCs, leaving only a cell-derived ECM coating attached to glass coverslips that can be used for functional assays. (**B**) Representative images of SMCs cultured 6 days on coverslips with a coating of POMA-FN, FN, gelatin or without a coating. All cells are positively stained for collagen type IV. After decellularization, deposited collagen type IV remained anchored only on POMA-FN and FN treated coverslips. Scale bar represents 100 µm. (**C**) Quantification of collagen type IV area deposited by SMC on coverslips with different coatings (POMA-FN, FN, gelatin or no coating). Shown is mean ± SEM, N=4-5. (**D**) Quantification of collagen type IV area deposited by SMC treated by different siRNA (siSham, siEMILIN1, siFBN1). Shown is mean ± SEM, N=5-7. Non-treated SMCs are set to one (dotted line).

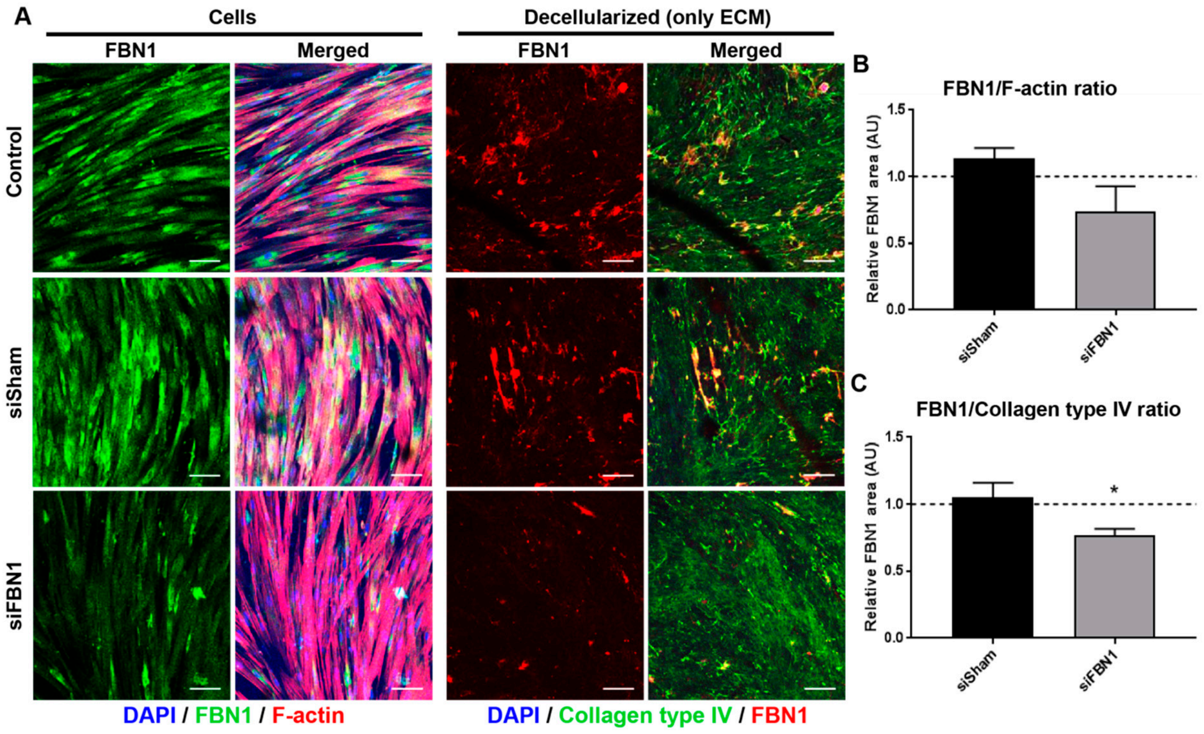

Supplemental Figure 4. (**A**) Representative Z-stacks of smooth muscle cell (SMCs) cultured for 6 days after siRNA transfection and after decellularization. Stained for FBN1 and either F-actin (cells) or collagen type IV (ECM). Scale bar represents 100 µm. (**B**) Quantification of FBN1 signal corrected for the amount of F-actin in siRNA treated SMCs. Shown is mean ± SEM, N=4. (**C**) Quantification of FBN1 signal corrected for the amount of collagen type IV present in SMC-derived ECM. Shown is mean ± SEM, N=5, *P<0,05. Non-treated SMC are set to one (dotted lines).

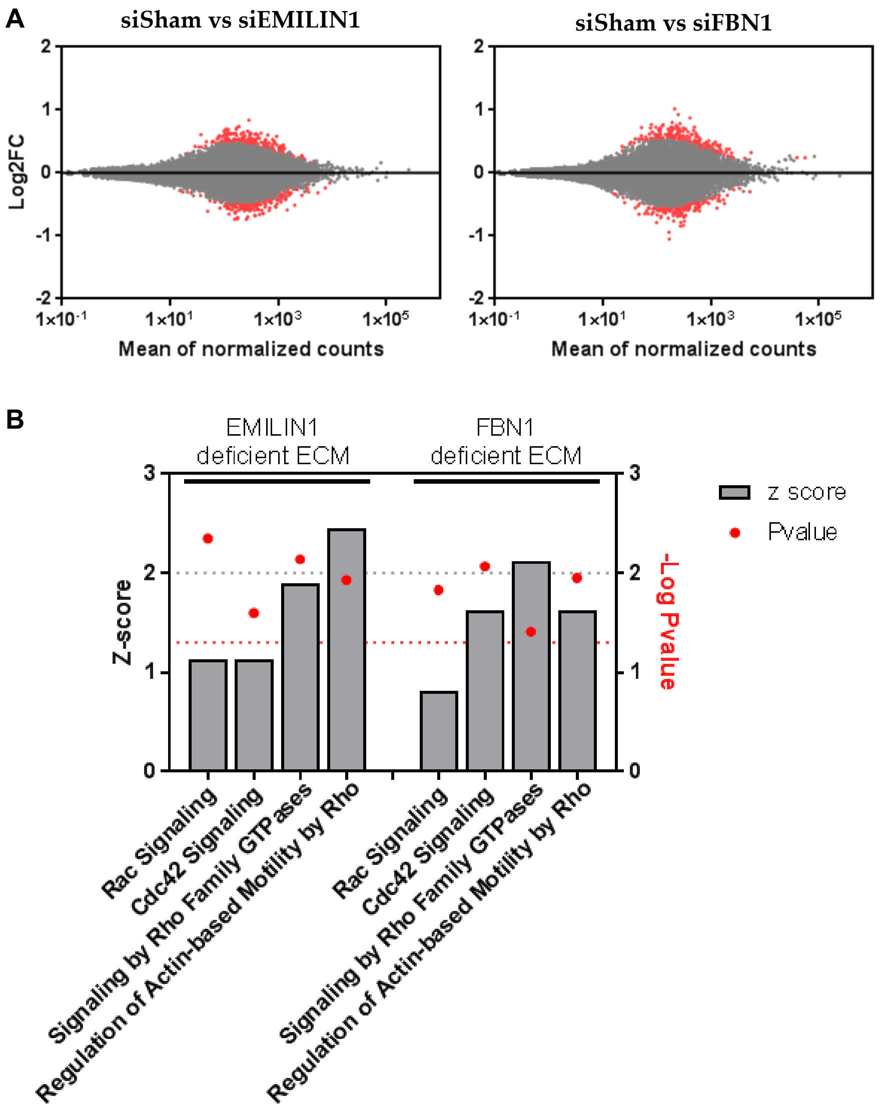

Supplemental Figure 5. (**A**) Visualization of differential gene expression in which Log2 fold change (FC) is plotted against the mean of normalized counts (MA-plot) in human umbilical vein endothelial cells (HUVECs) cultured on siSham extracellular matrix (ECM) vs. either EMILIN1-deficient or FBN1-deficient ECM. Gray dots represent non-differentially expressed genes, red dots represent differentially expressed genes ($P<0{,}05$, 481 and 474 genes for siEMILIN1 and siFBN1, respectively). RNA sequencing was performed on 3 samples per condition. (**B**) Z-score and P-value of pathways of the Rho GTPase family in HUVECs cultured on EMILIN1 or FBN1 deficient ECM.

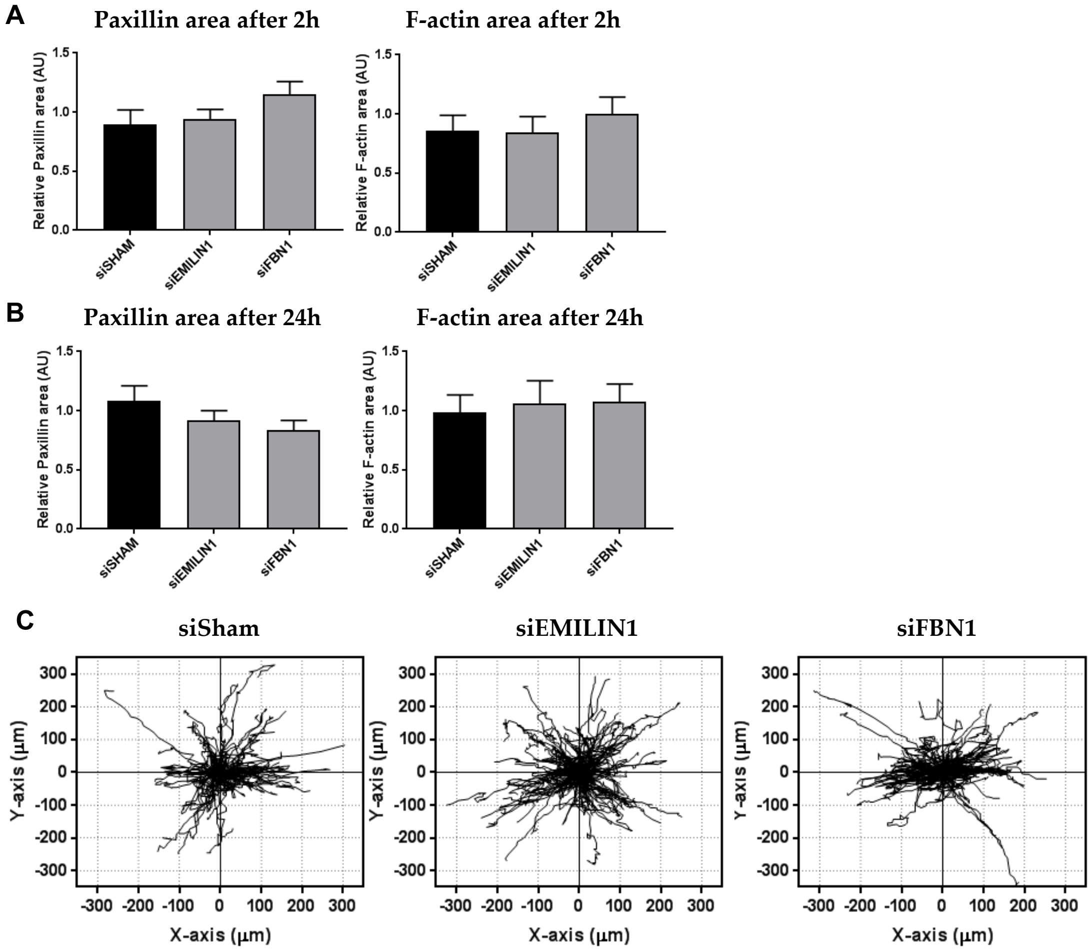

Supplemental Figure 6. (**A**) Paxillin area and F-actin area in HUVECs after 2 hours (not confluent) adhesion or after 24 hours (**B**) (confluent) adhesion on siRNA-treated ECM (siSham, siEMILIN1, siFBN1). Shown is mean ± SEM, N=4. (**C**) Representative migration plots showing tracks of individual HUVECs on siRNA treated ECM (siSham, siEMILIN1, siFBN1). Migration of HUVECs was traced overnight with confocal microscopy. N=<90 individual tracks per assay, N=4 assays.

Supplemental tables

Supplemental Table 1.

List of human fetal renal artery ECM protein abundance compared to mature

		Average percentage (LFQ intensity protein of interest/LFQ intensity total protein)							
**Protein name**	**Gene ID**	**Fetal**			**Mature**			**Fold change**	**P-value**
**Matrisome Core proteins**									
Collagens									
Collagen alpha-1(XII) chain	COL12A1	0,849	±	0,361	0,493	±	0,069	0,420	0,168
Collagen alpha-1(XIV) chain	COL14A1	1,720	±	0,597	0,934	±	0,420	0,457	0,136
Collagen alpha-1(XV) chain	COL15A1	0,011	±	0,010	0,126	±	0,016	-10,622	0,000
Collagen alpha-1(XVIII) chain	COL18A1	0,045	±	0,078	0,461	±	0,200	-9,261	0,028
Collagen alpha-1(I) chain	COL1A1	6,699	±	2,911	1,372	±	0,744	0,795	0,037
Collagen alpha-2(I) chain	COL1A2	5,865	±	1,674	1,022	±	0,510	0,826	0,009
Collagen alpha-1(XXI) chain	COL21A1	0,021	±	0,019	0,028	±	0,025	-0,333	0,724
Collagen alpha-1(III) chain	COL3A1	0,446	±	0,110	0,078	±	0,078	0,824	0,009
Collagen alpha-1(IV) chain	COL4A1	0,139	±	0,133	0,769	±	0,141	-4,530	0,005
Collagen alpha-2(IV) chain	COL4A2	0,197	±	0,180	1,587	±	0,073	-7,065	0,000
Collagen alpha-1(V) chain	COL5A1	0,103	±	0,084	ND			Unique in Fetal	0,099
Collagen alpha-1(VI) chain	COL6A1	8,087	±	1,603	8,076	±	2,485	0,001	0,995
Collagen alpha-2(VI) chain	COL6A2	4,450	±	0,924	3,500	±	0,267	0,214	0,162
Collagen alpha-3(VI) chain	COL6A3	17,098	±	3,652	11,292	±	3,411	0,340	0,115
Collagen alpha-6(VI) chain	COL6A6	0,128	±	0,037	ND	±		Unique in Fetal	0,004
Collagen alpha-1(VIII) chain	COL8A1	0,009	±	0,015	0,124	±	0,132	-13,424	0,208
Collagen alpha-2(VIII) chain	COL8A2	ND			0,014	±	0,017	Unique in Mature	0,237
Glycoproteins									
Adipocyte enhancer-binding protein 1	AEBP1	ND			0,004	±	0,004	Unique in Mature	0,161
Dermatopontin	DPT	0,166	±	0,191	0,680	±	0,534	-3,106	0,191
EGF-containing fibulin-like extracellular matrix protein 1	EFEMP1	0,004	±	0,004	0,017	±	0,007	-2,709	0,059
Elastin	ELN	0,052	±	0,029	0,086	±	0,075	-0,650	0,503
Elastin microfibrillar interface protein 1	EMILIN1	3,903	±	0,584	0,807	±	0,152	0,793	0,001
Elastin microfibrillar interface protein 2	EMILIN2	0,064	±	0,030	ND			Unique in Fetal	0,020
Elastin microfibrillar interface protein 3	EMILIN3	0,146	±	0,055	ND			Unique in Fetal	0,010
Fibulin-1	FBLN1	0,016	±	0,016	0,051	±	0,031	-2,207	0,156
Fibulin-2	FBLN2	0,024	±	0,021	0,058	±	0,017	-1,437	0,095
Fibulin-5	FBLN5	0,055	±	0,026	0,061	±	0,019	-0,100	0,782
Fibrillin-1	FBN1	11,846	±	3,003	1,077	±	0,158	0,909	0,003
Fibrillin-2	FBN2	4,652	±	1,773	0,009	±	0,008	0,998	0,011
Fibrinogen alpha chain	FGA	0,344	±	0,031	0,509	±	0,551	-0,481	0,631

Fibrinogen beta chain	FGB	0,271	±	0,082	0,717	±	0,448	-1,645	0,165
Fibrinogen gamma chain	FGG	0,444	±	0,158	0,971	±	0,558	-1,190	0,190
Fibronectin	FN1	1,618	±	0,587	4,944	±	2,358	-2,056	0,077
Hemicentin-2	HMCN2	0,027	±	0,030	ND			Unique in Fetal	0,199
Insulin-like growth factor-binding protein 7	IGFBP7	0,054	±	0,048	0,389	±	0,127	-6,210	0,013
Laminin subunit alpha-2	LAMA2	ND			0,001	±	0,001	Unique in Mature	0,467
Laminin subunit alpha-4	LAMA4	0,020	±	0,017	0,016	±	0,008	0,197	0,746
Laminin subunit alpha-5	LAMA5	0,265	±	0,162	1,280	±	0,183	-3,835	0,002
Laminin subunit beta-1	LAMB1	0,028	±	0,025	0,022	±	0,007	0,206	0,715
Laminin subunit beta-2	LAMB2	0,178	±	0,190	1,498	±	0,428	-7,396	0,008
Laminin subunit gamma-1	LAMC1	0,290	±	0,149	0,978	±	0,298	-2,375	0,023
Latent-transforming growth factor beta-binding protein 1	LTBP1	0,015	±	0,014	0,026	±	0,011	-0,793	0,327
Latent-transforming growth factor beta-binding protein 2	LTBP2	ND			0,009	±	0,009	Unique in Mature	0,167
Latent-transforming growth factor beta-binding protein 4	LTBP4	0,014	±	0,001	ND			Unique in Fetal	0,000
Matrilin-2	MATN2	0,003	±	0,005	0,002	±	0,002	0,238	0,835
Microfibrillar-associated protein 2	MFAP2	0,183	±	0,122	0,004	±	0,007	0,977	0,064
Microfibril-associated glycoprotein 4	MFAP4	0,006	±	0,006	0,070	±	0,052	-10,229	0,103
Microfibrillar-associated protein 5	MFAP5	0,250	±	0,165	0,011	±	0,010	0,957	0,066
Lactadherin	MFGE8	0,006	±	0,010	0,109	±	0,066	-17,229	0,055
Matrix Gla protein	MGP	0,011	±	0,020	0,059	±	0,042	-4,193	0,152
Multimerin-1	MMRN1	0,007	±	0,006	0,003	±	0,006	0,524	0,490
Nidogen-1	NID1	0,083	±	0,130	0,314	±	0,094	-2,774	0,067
Periostin	POSTN	6,340	±	1,582	4,502	±	1,914	0,290	0,269
Peroxidasin homolog	PXDN	0,007	±	0,002	0,001	±	0,003	0,779	0,057
Sushi repeat-containing protein	SRPX	0,002	±	0,004	0,010	±	0,009	-4,031	0,211
Transforming growth factor-beta-induced protein ig-h3	TGFBI	1,021	±	0,661	1,131	±	0,756	-0,108	0,858
Thrombospondin type-1 domain-containing protein 4	THSD4	0,009	±	0,004	0,003	±	0,004	0,621	0,186
Tubulointerstitial nephritis antigen-like	TINAGL1	0,023	±	0,040	0,348	±	0,245	-14,089	0,086
Tenascin	TNC	0,152	±	0,157	0,387	±	0,227	-1,544	0,215
Tenascin-X	TNXB	0,018	±	0,018	0,043	±	0,062	-1,413	0,533
Vitronectin	VTN	0,013	±	0,013	0,078	±	0,030	-4,849	0,028
von Willebrand factor A domain-containing protein 1	VWA1	0,003	±	0,003	0,018	±	0,011	-4,966	0,095
von Willebrand factor	VWF	0,065	±	0,050	0,041	±	0,036	0,371	0,535
Proteoglycans									

Aggrecan core protein	ACAN	0,015 ± 0,015	0,110 ± 0,103	-6,172	0,188
Biglycan	BGN	0,040 ± 0,045	0,293 ± 0,133	-6,289	0,036
Decorin	DCN	0,025 ± 0,026	0,460 ± 0,571	-17,715	0,257
Fibromodulin	FMOD	0,001 ± 0,001	0,003 ± 0,003	-3,066	0,379
Heparan sulfate proteoglycan core protein	HSPG2	1,154 ± 1,122	5,134 ± 2,022	-3,449	0,041
Lumican	LUM	0,088 ± 0,039	0,209 ± 0,228	-1,366	0,417
Osteoglycin	OGN	0,013 ± 0,022	0,040 ± 0,047	-2,124	0,417
Prolargin	PRELP	0,010 ± 0,018	0,149 ± 0,163	-13,465	0,216
Versican core protein	VCAN	0,158 ± 0,024	0,656 ± 0,864	-3,157	0,374
**Matrisome Associated proteins**					
ECM affiliated proteins					
Annexin A1	ANXA1	0,024 ± 0,012	0,070 ± 0,034	-1,949	0,091
Annexin A11	ANXA11	0,006 ± 0,006	0,027 ± 0,010	-3,695	0,038
Annexin A2	ANXA2	0,069 ± 0,008	0,023 ± 0,004	0,734	0,001
Annexin A4	ANXA4	0,014 ± 0,013	0,030 ± 0,014	-1,099	0,240
Annexin A5	ANXA5	0,075 ± 0,037	0,093 ± 0,040	-0,238	0,599
Annexin A6	ANXA6	0,082 ± 0,085	0,265 ± 0,050	-2,216	0,033
Annexin A7	ANXA7	0,003 ± 0,005	0,015 ± 0,003	-3,993	0,025
Complement C1q subcomponent subunit C	C1QC	0,002 ± 0,004	0,018 ± 0,028	-7,634	0,371
FRAS1-related extracellular matrix protein 2	FREM2	0,003 ± 0,004	ND	Unique in Fetal	0,266
Galectin-1	LGALS1	0,048 ± 0,043	0,035 ± 0,006	0,284	0,614
Protein ERGIC-53	LMAN1	0,002 ± 0,002	ND	Unique in Fetal	0,138
ECM regulating proteins					
Protein AMBP	AMBP	0,010 ± 0,017	0,325 ± 0,233	-32,642	0,080
Histidine-rich glycoprotein	HRG	0,002 ± 0,002	0,004 ± 0,003	-1,138	0,392
Serine protease HTRA1	HTRA1	0,003 ± 0,005	0,077 ± 0,055	-26,430	0,081
Inter-alpha-trypsin inhibitor heavy chain H1	ITIH1	0,276 ± 0,054	0,039 ± 0,017	0,860	0,002
Inter-alpha-trypsin inhibitor heavy chain H2	ITIH2	0,734 ± 0,074	0,002 ± 0,003	0,997	0,000
Inter-alpha-trypsin inhibitor heavy chain H3	ITIH3	0,011 ± 0,003	ND	Unique in Fetal	0,005
Inter-alpha-trypsin inhibitor heavy chain H5	ITIH5	0,001 ± 0,002	0,024 ± 0,012	-22,167	0,032
Alpha-1-antitrypsin	SERPINA1	0,004 ± 0,007	0,039 ± 0,017	-8,923	0,032
Antithrombin-III	SERPINC1	0,003 ± 0,005	0,005 ± 0,004	-0,504	0,711
Serpin H1	SERPINH1	0,042 ± 0,021	ND	Unique in Fetal	0,026
Protein-glutamine gamma-glutamyltransferase 2	TGM2	0,162 ± 0,117	0,477 ± 0,212	-1,947	0,088
Metalloproteinase inhibitor 3	TIMP3	0,008 ± 0,009	0,024 ± 0,022	-1,870	0,321

Secreted factors									
Angiopoietin-related protein 6	ANGPTL6	0,002	±	0,002	ND			Unique in Fetal	0,116
Protein S100-A11	S100A11	0,004	±	0,004	0,009	±	0,002	-1,137	0,128
Protein S100-A4	S100A4	ND			0,029	±	0,018	Unique in Mature	0,050
Protein S100-A7	S100A7	0,007	±	0,006	0,006	±	0,011	0,060	0,959
Protein S100-A8	S100A8	0,024	±	0,011	0,014	±	0,006	0,435	0,233
Protein S100-A9	S100A9	0,015	±	0,013	0,037	±	0,021	-1,528	0,194

ND = Not detected in the LC-MS/MS analysis

Supplemental Table 2.
Significant differentially expressed genes in non-targeting condition compared to HUVECs cultured on EMILIN1-deficient ECM

Symbol	ID	Log2FC	p value
FOXRED2	ENSG00000100350	0,84	1,63E-04
CD109	ENSG00000156535	-0,59	4,97E-04
MXRA7	ENSG00000182534	0,60	5,34E-04
PHLDB2	ENSG00000144824	-0,60	7,64E-04
ERAP2	ENSG00000164308	0,50	1,09E-03
LTBP1	ENSG00000049323	-0,56	1,30E-03
ARF3	ENSG00000134287	0,45	1,41E-03
ATP11A	ENSG00000068650	0,48	1,54E-03
SKIL	ENSG00000136603	-0,53	1,70E-03
ATM	ENSG00000149311	-0,66	1,75E-03
KNL1	ENSG00000137812	-0,47	1,81E-03
FKBP15	ENSG00000119321	-0,58	1,90E-03
PI4K2B	ENSG00000038210	-0,74	1,92E-03
MAP3K6	ENSG00000142733	0,74	2,04E-03
TM9SF3	ENSG00000077147	-0,51	2,04E-03
	ENSG00000257605	0,61	2,08E-03
NYNRIN	ENSG00000205978	-0,70	2,24E-03
ABR	ENSG00000159842	0,58	2,43E-03
IRF2BP2	ENSG00000168264	0,64	2,64E-03
ICE1	ENSG00000164151	-0,64	2,66E-03
CDC123	ENSG00000151465	-0,48	2,72E-03
MICU1	ENSG00000107745	0,63	2,74E-03
PANX1	ENSG00000110218	-0,73	2,76E-03
FCGRT	ENSG00000104870	-0,73	2,77E-03
TSC22D2	ENSG00000196428	0,56	2,81E-03
CCNL2	ENSG00000221978	-0,68	2,81E-03
	ENSG00000198327	0,69	2,87E-03
SEL1L3	ENSG00000091490	0,48	3,28E-03
ISG15	ENSG00000187608	-0,61	3,37E-03
MIF	ENSG00000240972	0,72	3,43E-03
GCC2	ENSG00000135968	-0,71	3,51E-03
PCDHGA11	ENSG00000253873	0,70	3,62E-03
MPP4	ENSG00000082126	0,70	3,71E-03
NIN	ENSG00000100503	-0,54	3,79E-03
B3GNT5	ENSG00000176597	-0,65	3,95E-03
FUBP3	ENSG00000107164	-0,68	4,39E-03
SNAP23	ENSG00000092531	-0,49	4,43E-03
LRP10	ENSG00000197324	0,40	4,49E-03
ZNF185	ENSG00000147394	0,42	4,50E-03
RAF1	ENSG00000132155	0,53	4,54E-03
MAP4	ENSG00000047849	-0,30	4,63E-03
UFL1	ENSG00000014123	0,61	4,70E-03
TCF3	ENSG00000071564	0,42	4,72E-03

MALAT1	ENSG00000251562	-0,32	4,85E-03
MED14	ENSG00000180182	-0,59	4,91E-03
RPUSD1	ENSG00000007376	0,62	5,15E-03
CNTROB	ENSG00000170037	0,54	5,32E-03
MERTK	ENSG00000153208	0,49	5,63E-03
ZNF839	ENSG00000022976	0,67	5,81E-03
CREB3	ENSG00000107175	0,60	5,88E-03
PLCB1	ENSG00000182621	0,63	6,10E-03
CASP3	ENSG00000164305	0,40	6,37E-03
C2orf47	ENSG00000162972	0,66	6,41E-03
CCAR2	ENSG00000158941	0,45	6,43E-03
PKI55	ENSG00000260804	0,67	6,46E-03
CASP10	ENSG00000003400	0,64	6,95E-03
GRB10	ENSG00000106070	0,42	7,02E-03
TOLLIP	ENSG00000078902	0,59	7,10E-03
HSPA4	ENSG00000170606	-0,40	7,18E-03
ERLEC1	ENSG00000068912	-0,57	7,29E-03
NRIP3	ENSG00000175352	0,59	7,35E-03
VPS51	ENSG00000149823	0,62	7,37E-03
G3BP1	ENSG00000145907	-0,36	7,48E-03
RHOJ	ENSG00000126785	0,42	7,49E-03
RPS21	ENSG00000171858	0,51	7,52E-03
RFXANK	ENSG00000064490	-0,65	7,61E-03
ITGA3	ENSG00000005884	0,35	7,71E-03
USP9X	ENSG00000124486	-0,42	8,23E-03
EFTUD2	ENSG00000108883	0,31	8,27E-03
NUTF2	ENSG00000102898	0,37	8,32E-03
GOT1	ENSG00000120053	-0,64	8,44E-03
SMIM12	ENSG00000163866	-0,54	8,54E-03
HSCB	ENSG00000100209	0,55	8,83E-03
AKAP12	ENSG00000131016	-0,24	8,91E-03
CHN1	ENSG00000128656	0,64	9,10E-03
ANKIB1	ENSG00000001629	-0,59	9,35E-03
HOMER3	ENSG00000051128	0,64	9,57E-03
NARS	ENSG00000134440	-0,38	9,59E-03
ETS1	ENSG00000134954	-0,44	9,77E-03
UNKL	ENSG00000059145	0,62	9,88E-03
COX11	ENSG00000166260	0,55	9,91E-03
ACIN1	ENSG00000100813	0,38	1,00E-02
ZNF589	ENSG00000164048	0,62	1,01E-02
SLC38A9	ENSG00000177058	0,63	1,04E-02
ELMO1	ENSG00000155849	0,58	1,04E-02
RAP2A	ENSG00000125249	-0,58	1,06E-02
METTL6	ENSG00000206562	0,62	1,06E-02
LRRC8C	ENSG00000171488	0,31	1,07E-02
CARD6	ENSG00000132357	-0,57	1,08E-02

FAAP100	ENSG00000185504	0,55	1,10E-02
SIDT2	ENSG00000149577	0,59	1,10E-02
PAQR7	ENSG00000182749	0,62	1,11E-02
MMRN2	ENSG00000173269	-0,26	1,11E-02
EIF4B	ENSG00000063046	-0,42	1,11E-02
HUWE1	ENSG00000086758	-0,28	1,12E-02
MCUR1	ENSG00000050393	0,55	1,14E-02
LRP12	ENSG00000147650	0,62	1,14E-02
PNPLA8	ENSG00000135241	0,55	1,14E-02
BRAF	ENSG00000157764	-0,62	1,16E-02
SRPX	ENSG00000101955	0,45	1,16E-02
FASTKD2	ENSG00000118246	0,45	1,18E-02
LPAR6	ENSG00000139679	0,59	1,22E-02
SETD3	ENSG00000183576	-0,59	1,25E-02
TMEM115	ENSG00000126062	0,49	1,27E-02
INO80	ENSG00000128908	0,53	1,29E-02
PRKD3	ENSG00000115825	-0,46	1,29E-02
MAPK14	ENSG00000112062	-0,50	1,33E-02
	ENSG00000271430	0,61	1,33E-02
MRPL20	ENSG00000242485	0,55	1,34E-02
LTV1	ENSG00000135521	-0,60	1,35E-02
	ENSG00000108270	-0,51	1,36E-02
SPRYD3	ENSG00000167778	-0,56	1,36E-02
SLC5A6	ENSG00000138074	-0,57	1,36E-02
MCRIP2	ENSG00000172366	0,50	1,37E-02
PCNX3	ENSG00000197136	0,48	1,37E-02
FDFT1	ENSG00000079459	-0,43	1,39E-02
PHKA1	ENSG00000067177	0,60	1,39E-02
SNX12	ENSG00000147164	0,45	1,41E-02
RPN2	ENSG00000118705	-0,26	1,43E-02
TPD52L2	ENSG00000101150	-0,36	1,43E-02
CHTOP	ENSG00000160679	-0,60	1,43E-02
ANKRD12	ENSG00000101745	-0,58	1,45E-02
WDR43	ENSG00000163811	-0,37	1,46E-02
PRPF39	ENSG00000185246	-0,60	1,46E-02
NOP58	ENSG00000055044	-0,45	1,46E-02
NTN4	ENSG00000074527	0,31	1,46E-02
LOC105372481	ENSG00000142396	0,57	1,49E-02
RBM39	ENSG00000131051	0,32	1,50E-02
OAZ2	ENSG00000180304	-0,55	1,51E-02
HMOX1	ENSG00000100292	0,43	1,54E-02
ALDH1L2	ENSG00000136010	0,59	1,54E-02
SNRPD2	ENSG00000125743	0,48	1,54E-02
TFDP1	ENSG00000198176	-0,39	1,55E-02
PRPF19	ENSG00000110107	0,40	1,57E-02
MYLK	ENSG00000065534	-0,56	1,58E-02

RFX2	ENSG00000087903	0,59	1,59E-02
EFNA5	ENSG00000184349	-0,59	1,59E-02
SUMO1	ENSG00000116030	-0,59	1,60E-02
SSFA2	ENSG00000138434	-0,41	1,60E-02
IFNAR2	ENSG00000159110	0,56	1,61E-02
DEGS1	ENSG00000143753	0,38	1,61E-02
FLOT2	ENSG00000132589	0,48	1,67E-02
THUMPD2	ENSG00000138050	0,58	1,68E-02
FRYL	ENSG00000075539	-0,54	1,69E-02
SULF2	ENSG00000196562	-0,46	1,69E-02
WASL	ENSG00000106299	0,45	1,74E-02
RINT1	ENSG00000135249	-0,54	1,75E-02
CDC42	ENSG00000070831	0,25	1,75E-02
DNAJC9	ENSG00000213551	-0,58	1,75E-02
KIAA0907	ENSG00000132680	0,52	1,76E-02
PRCC	ENSG00000143294	-0,50	1,77E-02
APEH	ENSG00000164062	0,53	1,78E-02
MAP3K4	ENSG00000085511	0,50	1,81E-02
PFKP	ENSG00000067057	0,28	1,83E-02
KDELR2	ENSG00000136240	-0,35	1,85E-02
TRAK1	ENSG00000182606	0,40	1,87E-02
HTRA1	ENSG00000166033	-0,37	1,87E-02
TULP3	ENSG00000078246	0,50	1,87E-02
CCNG1	ENSG00000113328	-0,42	1,88E-02
DNAJC2	ENSG00000105821	-0,57	1,90E-02
SPAG9	ENSG00000008294	-0,42	1,90E-02
CD151	ENSG00000177697	-0,32	1,91E-02
DDX39A	ENSG00000123136	0,40	1,91E-02
BACE1	ENSG00000186318	-0,52	1,91E-02
IGFBP7	ENSG00000163453	-0,30	1,92E-02
ZNF768	ENSG00000169957	0,54	1,92E-02
KIAA1217	ENSG00000120549	0,54	1,95E-02
PRKCI	ENSG00000163558	-0,57	1,99E-02
FAM111A	ENSG00000166801	-0,50	2,00E-02
IDS	ENSG00000010404	-0,46	2,02E-02
SNRPC	ENSG00000124562	0,42	2,04E-02
DUSP23	ENSG00000158716	-0,57	2,05E-02
SLC29A1	ENSG00000112759	0,23	2,06E-02
NRGN	ENSG00000154146	0,54	2,07E-02
MTFR1L	ENSG00000117640	-0,57	2,08E-02
ZMAT3	ENSG00000172667	0,41	2,09E-02
GPR180	ENSG00000152749	-0,51	2,13E-02
ATP6V1G1	ENSG00000136888	-0,54	2,13E-02
NIP7	ENSG00000132603	0,48	2,13E-02
WAPL	ENSG00000062650	-0,36	2,13E-02
MRPL44	ENSG00000135900	0,54	2,14E-02

HNRNPH3	ENSG00000096746	-0,29	2,14E-02
PEX16	ENSG00000121680	0,56	2,14E-02
LDOC1L	ENSG00000188636	-0,55	2,14E-02
RHOBTB3	ENSG00000164292	-0,39	2,14E-02
TBPL1	ENSG00000028839	-0,52	2,15E-02
USP37	ENSG00000135913	0,55	2,15E-02
NAA25	ENSG00000111300	-0,54	2,17E-02
C1GALT1	ENSG00000106392	-0,56	2,19E-02
SLU7	ENSG00000164609	0,54	2,21E-02
AHSA1	ENSG00000100591	0,35	2,22E-02
GAS2L1	ENSG00000185340	0,52	2,23E-02
GALNT7	ENSG00000109586	-0,42	2,24E-02
RMND5A	ENSG00000153561	-0,52	2,24E-02
ARGLU1	ENSG00000134884	-0,51	2,24E-02
BCAM	ENSG00000187244	0,54	2,25E-02
POLE3	ENSG00000148229	0,36	2,25E-02
RANBP2	ENSG00000153201	-0,35	2,25E-02
ANAPC13	ENSG00000129055	0,52	2,25E-02
SNRNP200	ENSG00000144028	0,26	2,26E-02
PREB	ENSG00000138073	-0,52	2,27E-02
TMEM106A	ENSG00000184988	0,56	2,29E-02
PIKFYVE	ENSG00000115020	0,43	2,29E-02
RFTN2	ENSG00000162944	0,42	2,34E-02
DCAF1	ENSG00000145041	0,43	2,36E-02
MAF1	ENSG00000179632	-0,54	2,36E-02
C6orf48	ENSG00000204387	0,51	2,37E-02
EI24	ENSG00000149547	0,24	2,41E-02
DENND5A	ENSG00000184014	0,30	2,41E-02
KIF21A	ENSG00000139116	-0,52	2,42E-02
TRMT10C	ENSG00000174173	-0,55	2,43E-02
ELF2	ENSG00000109381	-0,55	2,43E-02
ADTRP	ENSG00000111863	-0,55	2,44E-02
SMPDL3A	ENSG00000172594	0,50	2,44E-02
RAD23B	ENSG00000119318	0,30	2,44E-02
LAMA4	ENSG00000112769	-0,25	2,45E-02
ERH	ENSG00000100632	0,32	2,46E-02
CASC15	ENSG00000272168	0,41	2,48E-02
CPD	ENSG00000108582	-0,35	2,48E-02
	ENSG00000233137	-0,54	2,51E-02
RPSA	ENSG00000168028	0,25	2,51E-02
NSFL1C	ENSG00000088833	0,33	2,52E-02
ITPR1	ENSG00000150995	-0,55	2,53E-02
SOS2	ENSG00000100485	-0,55	2,53E-02
FBXO9	ENSG00000112146	-0,55	2,55E-02
MIER1	ENSG00000198160	-0,42	2,55E-02
CREBBP	ENSG00000005339	0,43	2,56E-02

RNF187	ENSG00000168159	-0,52	2,56E-02
PRKCD	ENSG00000163932	-0,55	2,58E-02
SH3BGRL3	ENSG00000142669	0,22	2,58E-02
H2AFX	ENSG00000188486	-0,41	2,58E-02
VPS41	ENSG00000006715	0,38	2,60E-02
MAT2B	ENSG00000038274	-0,50	2,64E-02
VASH1	ENSG00000071246	0,52	2,66E-02
TAF1B	ENSG00000115750	0,52	2,68E-02
TPI1	ENSG00000111669	0,23	2,69E-02
DERL1	ENSG00000136986	-0,44	2,70E-02
SGMS2	ENSG00000164023	0,32	2,72E-02
TAX1BP1	ENSG00000106052	0,34	2,72E-02
FAM107A	ENSG00000168309	0,39	2,74E-02
RPS6KA1	ENSG00000117676	0,54	2,76E-02
ERI1	ENSG00000104626	-0,54	2,76E-02
SLC3A2	ENSG00000168003	0,40	2,79E-02
FAM220A	ENSG00000178397	0,53	2,79E-02
RIC1	ENSG00000107036	-0,51	2,80E-02
SNU13	ENSG00000100138	0,41	2,82E-02
SKA1	ENSG00000154839	-0,49	2,82E-02
XRN1	ENSG00000114127	-0,49	2,85E-02
USP15	ENSG00000135655	-0,42	2,85E-02
SKP1	ENSG00000113558	-0,39	2,87E-02
	ENSG00000188971	-0,39	2,87E-02
TRIP11	ENSG00000100815	-0,47	2,88E-02
TMEM218	ENSG00000150433	0,52	2,88E-02
TEAD4	ENSG00000197905	0,46	2,90E-02
NEU1	ENSG00000204386	0,49	2,92E-02
SLC1A4	ENSG00000115902	-0,53	2,92E-02
PNRC2	ENSG00000189266	-0,39	2,93E-02
	ENSG00000141720	0,41	2,94E-02
RBAK	ENSG00000146587	0,53	2,94E-02
SPECC1L	ENSG00000100014	0,45	2,95E-02
CDV3	ENSG00000091527	0,23	2,96E-02
SRPX2	ENSG00000102359	-0,36	2,96E-02
KIAA0930	ENSG00000100364	-0,53	2,97E-02
RPS6KB1	ENSG00000108443	0,46	2,98E-02
ZHX1	ENSG00000165156	-0,43	3,00E-02
DNAJC21	ENSG00000168724	-0,46	3,03E-02
CLSTN1	ENSG00000171603	0,27	3,03E-02
RASGRP2	ENSG00000068831	0,32	3,03E-02
RAB18	ENSG00000099246	-0,42	3,04E-02
NDUFB7	ENSG00000099795	0,52	3,06E-02
GPR107	ENSG00000148358	0,33	3,08E-02
CAMTA2	ENSG00000108509	0,49	3,08E-02
CTSC	ENSG00000109861	0,27	3,09E-02

FRY	ENSG00000073910	0,52	3,09E-02
RAB5A	ENSG00000144566	0,33	3,09E-02
MPP1	ENSG00000130830	0,53	3,11E-02
DTYMK	ENSG00000168393	-0,44	3,11E-02
CAPN15	ENSG00000103326	0,45	3,12E-02
PPP2CA	ENSG00000113575	0,25	3,12E-02
TUBG1	ENSG00000131462	0,39	3,13E-02
MRPL18	ENSG00000112110	0,46	3,14E-02
	ENSG00000218283	0,53	3,15E-02
BNIP3	ENSG00000176171	0,50	3,15E-02
R3HDM1	ENSG00000048991	-0,50	3,18E-02
TRPC4	ENSG00000133107	0,41	3,18E-02
RUNX1	ENSG00000159216	-0,49	3,18E-02
WASF3	ENSG00000132970	0,52	3,19E-02
FOCAD	ENSG00000188352	-0,46	3,20E-02
SLC7A6OS	ENSG00000103061	0,51	3,22E-02
RPL26	ENSG00000161970	-0,30	3,22E-02
NANP	ENSG00000170191	-0,52	3,23E-02
SEC24B	ENSG00000138802	0,34	3,25E-02
NUBP1	ENSG00000103274	0,52	3,26E-02
TUBA1C	ENSG00000167553	0,19	3,27E-02
	ENSG00000117289	-0,47	3,31E-02
ARHGAP11A	ENSG00000198826	-0,34	3,31E-02
STAT2	ENSG00000170581	-0,52	3,32E-02
UBE2D1	ENSG00000072401	-0,51	3,33E-02
MRC2	ENSG00000011028	-0,48	3,36E-02
ALS2	ENSG00000003393	-0,47	3,37E-02
CCSER2	ENSG00000107771	0,34	3,38E-02
TWISTNB	ENSG00000105849	0,35	3,39E-02
RNH1	ENSG00000023191	0,39	3,40E-02
FJX1	ENSG00000179431	0,47	3,40E-02
PDE3A	ENSG00000172572	-0,49	3,41E-02
PAPOLG	ENSG00000115421	-0,52	3,43E-02
ZNF521	ENSG00000198795	0,41	3,43E-02
RUVBL2	ENSG00000183207	0,40	3,45E-02
CDCA5	ENSG00000146670	0,40	3,47E-02
CCDC84	ENSG00000186166	0,51	3,47E-02
DGKA	ENSG00000065357	0,45	3,48E-02
PPP1R2	ENSG00000184203	0,43	3,48E-02
KIAA1109	ENSG00000138688	0,38	3,48E-02
EIF1AX	ENSG00000173674	0,34	3,49E-02
PEBP1	ENSG00000089220	-0,41	3,49E-02
	ENSG00000260336	0,51	3,50E-02
COL4A3BP	ENSG00000113163	-0,50	3,51E-02
CNPY2	ENSG00000257727	0,50	3,51E-02
FOXM1	ENSG00000111206	0,27	3,51E-02

LRRC41	ENSG00000132128	-0,39	3,52E-02
FLII	ENSG00000177731	0,31	3,52E-02
ACSL4	ENSG00000068366	-0,32	3,53E-02
MARCH6	ENSG00000145495	-0,35	3,53E-02
SPATS2	ENSG00000123352	0,35	3,55E-02
RPN1	ENSG00000163902	0,23	3,55E-02
SUPT20H	ENSG00000102710	-0,47	3,56E-02
TRA2A	ENSG00000164548	-0,46	3,57E-02
DICER1	ENSG00000100697	-0,40	3,59E-02
CBR3	ENSG00000159231	0,49	3,60E-02
TTK	ENSG00000112742	-0,43	3,62E-02
CAD	ENSG00000084774	0,30	3,62E-02
PDE8A	ENSG00000073417	-0,49	3,68E-02
CCDC88A	ENSG00000115355	-0,36	3,69E-02
FURIN	ENSG00000140564	0,29	3,69E-02
TCP11L1	ENSG00000176148	-0,45	3,70E-02
CIAO1	ENSG00000144021	-0,46	3,71E-02
ZBTB17	ENSG00000116809	-0,51	3,71E-02
OSTC	ENSG00000198856	-0,44	3,72E-02
NRP1	ENSG00000099250	-0,24	3,73E-02
PCYOX1L	ENSG00000145882	-0,45	3,74E-02
MTMR2	ENSG00000087053	-0,40	3,77E-02
TMEM25	ENSG00000149582	-0,39	3,77E-02
GPR176	ENSG00000166073	-0,40	3,78E-02
SSBP1	ENSG00000106028	-0,40	3,78E-02
SCP2	ENSG00000116171	0,41	3,78E-02
	ENSG00000237181	0,31	3,79E-02
NUP107	ENSG00000111581	0,41	3,80E-02
COX8A	ENSG00000176340	0,47	3,80E-02
	ENSG00000239665	-0,32	3,80E-02
HHIP-AS1	ENSG00000248890	0,39	3,81E-02
GPX1	ENSG00000233276	0,39	3,83E-02
RCOR1	ENSG00000089902	0,37	3,84E-02
GNG5	ENSG00000174021	0,30	3,88E-02
FAM206A	ENSG00000119328	0,48	3,88E-02
MAN2A2	ENSG00000196547	0,41	3,89E-02
ZNF267	ENSG00000185947	-0,50	3,89E-02
SYNE2	ENSG00000054654	-0,38	3,90E-02
ZFHX4	ENSG00000091656	-0,30	3,90E-02
IGHMBP2	ENSG00000132740	-0,44	3,90E-02
PLEKHB2	ENSG00000115762	0,29	3,91E-02
RSBN1L	ENSG00000187257	-0,51	3,91E-02
TIGAR	ENSG00000078237	-0,44	3,91E-02
SRP9	ENSG00000143742	-0,34	3,93E-02
DMTN	ENSG00000158856	0,50	3,94E-02
SSX2IP	ENSG00000117155	0,50	3,94E-02

PPID	ENSG00000171497	0,45	3,94E-02
SRGAP2B	ENSG00000196369	-0,46	3,95E-02
MSH6	ENSG00000116062	-0,42	3,95E-02
MID1	ENSG00000101871	-0,46	3,95E-02
ENTPD4	ENSG00000197217	-0,45	3,95E-02
CENPF	ENSG00000117724	-0,26	3,98E-02
ORC3	ENSG00000135336	0,47	3,99E-02
CEP78	ENSG00000148019	0,40	3,99E-02
SETD2	ENSG00000181555	-0,34	4,02E-02
SNRNP35	ENSG00000184209	0,50	4,02E-02
CDR2L	ENSG00000109089	0,40	4,04E-02
IFI16	ENSG00000163565	-0,26	4,04E-02
TRIB1	ENSG00000173334	-0,50	4,07E-02
SAPCD2	ENSG00000186193	0,46	4,07E-02
NUDT3	ENSG00000272325	-0,35	4,10E-02
	ENSG00000241717	0,47	4,12E-02
LACTB2	ENSG00000147592	0,47	4,13E-02
SEC61G	ENSG00000132432	-0,47	4,13E-02
TSPAN18	ENSG00000157570	0,47	4,15E-02
SMAP1	ENSG00000112305	-0,49	4,15E-02
PKN3	ENSG00000160447	0,45	4,16E-02
SDE2	ENSG00000143751	-0,50	4,17E-02
GLMP	ENSG00000198715	-0,50	4,17E-02
DENND6A	ENSG00000174839	-0,50	4,17E-02
MYO1E	ENSG00000157483	-0,42	4,18E-02
DTL	ENSG00000143476	-0,48	4,18E-02
ZNF664	ENSG00000179195	0,37	4,18E-02
AP3S2	ENSG00000157823	0,49	4,20E-02
PSCA	ENSG00000167653	-0,29	4,21E-02
MAD2L1	ENSG00000164109	-0,47	4,22E-02
FBXW11	ENSG00000072803	-0,45	4,22E-02
ZNF800	ENSG00000048405	0,45	4,24E-02
DDX24	ENSG00000089737	-0,33	4,25E-02
PSD3	ENSG00000156011	-0,50	4,26E-02
HNRNPA1	ENSG00000135486	-0,18	4,26E-02
SPIN4	ENSG00000186767	0,50	4,27E-02
LUC7L3	ENSG00000108848	0,29	4,27E-02
FAM102B	ENSG00000162636	-0,44	4,27E-02
UFC1	ENSG00000143222	-0,42	4,28E-02
CS	ENSG00000062485	0,29	4,30E-02
RPS27A	ENSG00000143947	-0,24	4,33E-02
CRACR2A	ENSG00000130038	0,49	4,33E-02
TES	ENSG00000135269	-0,47	4,33E-02
TBCA	ENSG00000171530	0,43	4,34E-02
HIBCH	ENSG00000198130	0,49	4,36E-02
NAB1	ENSG00000138386	-0,49	4,37E-02

LMTK2	ENSG00000164715	-0,48	4,37E-02
NECAP2	ENSG00000157191	-0,45	4,38E-02
ATP5G3	ENSG00000154518	0,33	4,42E-02
MEG3	ENSG00000214548	-0,48	4,43E-02
ZNF512B	ENSG00000196700	0,44	4,44E-02
SMAD3	ENSG00000166949	-0,31	4,46E-02
TIMP1	ENSG00000102265	0,37	4,48E-02
FANCD2	ENSG00000144554	-0,44	4,48E-02
PARP9	ENSG00000138496	-0,49	4,50E-02
FAM43A	ENSG00000185112	0,26	4,51E-02
FPGS	ENSG00000136877	0,42	4,53E-02
NDUFA6	ENSG00000184983	0,43	4,54E-02
BACH1	ENSG00000156273	-0,49	4,56E-02
CHTF8	ENSG00000168802	0,45	4,56E-02
MITD1	ENSG00000158411	-0,49	4,56E-02
MAPK12	ENSG00000188130	-0,47	4,57E-02
GIMAP4	ENSG00000133574	-0,48	4,57E-02
CLIP3	ENSG00000105270	0,47	4,57E-02
NEDD4L	ENSG00000049759	0,40	4,59E-02
RDH11	ENSG00000072042	0,33	4,60E-02
PLEKHG3	ENSG00000126822	-0,36	4,60E-02
C2CD3	ENSG00000168014	-0,42	4,61E-02
MIR568	ENSG00000259976	-0,28	4,62E-02
TOE1	ENSG00000132773	-0,49	4,63E-02
UBAP2	ENSG00000137073	-0,45	4,64E-02
CHST3	ENSG00000122863	0,46	4,64E-02
CYP2R1	ENSG00000186104	0,48	4,64E-02
FSCN1	ENSG00000075618	0,25	4,65E-02
HAX1	ENSG00000143575	0,44	4,66E-02
ABHD2	ENSG00000140526	0,32	4,67E-02
LYN	ENSG00000254087	-0,49	4,68E-02
PRDX4	ENSG00000123131	0,38	4,69E-02
CKS2	ENSG00000123975	-0,45	4,69E-02
POLR2E	ENSG00000099817	0,32	4,70E-02
EIF3H	ENSG00000147677	-0,32	4,70E-02
SERF2	ENSG00000140264	0,30	4,70E-02
EIF2AK1	ENSG00000086232	0,28	4,71E-02
ERMAP	ENSG00000164010	0,41	4,71E-02
DEPDC1	ENSG00000024526	0,33	4,72E-02
HEG1	ENSG00000173706	0,27	4,74E-02
ZNF383	ENSG00000188283	0,43	4,75E-02
C9orf40	ENSG00000135045	0,48	4,76E-02
WDR26	ENSG00000162923	-0,37	4,76E-02
SGSH	ENSG00000181523	0,43	4,76E-02
PRRG1	ENSG00000130962	-0,48	4,77E-02
FAM168B	ENSG00000152102	0,30	4,77E-02

DLG1	ENSG00000075711	-0,41	4,78E-02
PKNOX1	ENSG00000160199	-0,49	4,79E-02
REC8	ENSG00000100918	0,48	4,80E-02
DYNLL2	ENSG00000264364	0,38	4,81E-02
PRELID3B	ENSG00000101166	0,35	4,82E-02
SLC25A30	ENSG00000174032	0,48	4,82E-02
TMEM127	ENSG00000135956	-0,41	4,83E-02
ZNF697	ENSG00000143067	-0,48	4,83E-02
SERTAD3	ENSG00000167565	0,48	4,86E-02
GATC	ENSG00000257218	-0,48	4,86E-02
PAQR4	ENSG00000162073	0,45	4,86E-02
COL4A1	ENSG00000187498	-0,16	4,87E-02
HARS2	ENSG00000112855	-0,48	4,87E-02
NUP85	ENSG00000125450	-0,46	4,88E-02
SART3	ENSG00000075856	0,37	4,89E-02
CRTAP	ENSG00000170275	0,28	4,91E-02
PAM	ENSG00000145730	0,26	4,91E-02
CCDC137	ENSG00000185298	-0,48	4,91E-02
NONO	ENSG00000147140	0,20	4,92E-02
SUCO	ENSG00000094975	-0,46	4,93E-02
SLC4A2	ENSG00000164889	0,32	4,93E-02
PHLPP2	ENSG00000040199	0,48	4,96E-02
UIMC1	ENSG00000087206	0,42	4,99E-02
BIRC5	ENSG00000089685	-0,34	4,99E-02

Supplemental Table 3.
Significant differentially expressed genes in non-targeting condition compared to HUVECs cultured on FBN1-deficient ECM

Symbol	ID	Log2FC	p value
IRF2BP2	ENSG00000168264	0,88	7,30E-06
PANX1	ENSG00000110218	-1,05	1,07E-04
PKI55	ENSG00000260804	1,02	2,02E-04
MBD3	ENSG00000071655	-0,83	2,60E-04
FCGRT	ENSG00000104870	-0,94	3,65E-04
ZNF800	ENSG00000048405	0,93	4,16E-04
HECTD1	ENSG00000092148	0,44	5,25E-04
MAF1	ENSG00000179632	-0,84	7,61E-04
PAWR	ENSG00000177425	0,52	9,07E-04
FASTKD2	ENSG00000118246	0,68	1,22E-03
INO80	ENSG00000128908	0,73	1,38E-03
BNIP3	ENSG00000176171	0,80	1,49E-03
GPATCH4	ENSG00000160818	0,61	1,66E-03
NNMT	ENSG00000166741	-0,60	1,78E-03
TMED9	ENSG00000184840	-0,49	1,89E-03
PLAUR	ENSG00000011422	-0,60	2,17E-03
FUBP3	ENSG00000107164	-0,76	2,40E-03
ERG	ENSG00000157554	0,42	2,51E-03
ND4L	ENSG00000212907	0,33	3,04E-03
PCBD1	ENSG00000166228	0,78	3,11E-03
KIAA0319L	ENSG00000142687	-0,68	3,14E-03
EIF4B	ENSG00000063046	-0,56	3,17E-03
NOP10	ENSG00000182117	-0,79	3,40E-03
CKAP2L	ENSG00000169607	0,58	3,45E-03
PHLDB2	ENSG00000144824	-0,55	3,46E-03
TMSB4X	ENSG00000205542	-0,30	3,46E-03
H2AFZ	ENSG00000164032	-0,40	3,47E-03
RN7SK	ENSG00000202198	-0,44	3,76E-03
MAPK11	ENSG00000185386	-0,79	3,78E-03
ATP8	ENSG00000228253	0,49	3,80E-03
HTRA1	ENSG00000166033	-0,53	3,92E-03
WASF3	ENSG00000132970	0,76	3,98E-03
IFNAR2	ENSG00000159110	0,73	4,08E-03
NTN4	ENSG00000074527	0,43	4,16E-03
ZFYVE16	ENSG00000039319	0,56	4,19E-03
CKS2	ENSG00000123975	-0,70	4,28E-03
ERCC4	ENSG00000175595	0,77	4,32E-03
OTUD5	ENSG00000068308	0,73	4,45E-03
ERMAP	ENSG00000164010	0,72	4,50E-03
RCN3	ENSG00000142552	-0,56	4,67E-03
KLHDC10	ENSG00000128607	0,59	4,74E-03
CHSY3	ENSG00000198108	0,77	4,80E-03
PRKCI	ENSG00000163558	-0,76	5,11E-03

TRIQK	ENSG00000205133	0,75	5,21E-03
CD151	ENSG00000177697	-0,45	5,33E-03
MAFG	ENSG00000197063	-0,69	5,34E-03
ISG15	ENSG00000187608	-0,66	5,37E-03
SKAP2	ENSG00000005020	0,65	5,42E-03
DDX6	ENSG00000110367	0,39	5,48E-03
RPS6KB1	ENSG00000108443	0,70	5,54E-03
PKIA	ENSG00000171033	0,51	5,64E-03
LRP12	ENSG00000147650	0,73	5,70E-03
NMT2	ENSG00000152465	0,51	5,87E-03
USP19	ENSG00000172046	-0,72	5,90E-03
ARF3	ENSG00000134287	0,46	6,08E-03
BDP1	ENSG00000145734	0,54	6,21E-03
MMRN2	ENSG00000173269	-0,36	6,22E-03
MXRA7	ENSG00000182534	0,58	6,32E-03
MCM7	ENSG00000166508	-0,45	6,34E-03
MAML2	ENSG00000184384	0,70	6,60E-03
SPPL3	ENSG00000157837	0,71	6,76E-03
ABHD2	ENSG00000140526	0,42	7,05E-03
	ENSG00000271430	0,73	7,19E-03
	ENSG00000225630	0,48	7,35E-03
ELMO1	ENSG00000155849	0,71	7,47E-03
WASL	ENSG00000106299	0,59	7,69E-03
CCNYL1	ENSG00000163249	-0,58	7,69E-03
GLMP	ENSG00000198715	-0,72	7,91E-03
SFXN1	ENSG00000164466	0,45	8,08E-03
APEH	ENSG00000164062	0,66	8,12E-03
PLCB1	ENSG00000182621	0,66	8,14E-03
SAP18	ENSG00000150459	-0,58	8,16E-03
THUMPD2	ENSG00000138050	0,71	8,24E-03
RPUSD1	ENSG00000007376	0,69	8,24E-03
PPID	ENSG00000171497	0,65	8,30E-03
GSPT1	ENSG00000103342	0,38	8,40E-03
STRIP2	ENSG00000128578	0,62	8,42E-03
TAF3	ENSG00000165632	0,69	8,50E-03
VASH1	ENSG00000071246	0,72	8,56E-03
FAM206A	ENSG00000119328	0,69	8,62E-03
NRK	ENSG00000123572	0,71	8,64E-03
CAMK2D	ENSG00000145349	0,68	8,67E-03
PREB	ENSG00000138073	-0,65	8,70E-03
LYPD1	ENSG00000150551	-0,59	8,78E-03
MIOS	ENSG00000164654	0,66	8,93E-03
ELOVL5	ENSG00000012660	0,44	9,05E-03
TOE1	ENSG00000132773	-0,71	9,19E-03
ZDHHC7	ENSG00000153786	-0,68	9,39E-03
LIFR	ENSG00000113594	0,70	9,64E-03

CEP78	ENSG00000148019	0,52	9,71E-03
CCSER2	ENSG00000107771	0,64	9,82E-03
BIRC5	ENSG00000089685	-0,55	9,97E-03
LUC7L3	ENSG00000108848	0,46	1,00E-02
COPS5	ENSG00000121022	-0,68	1,02E-02
GIMAP4	ENSG00000133574	-0,67	1,05E-02
TM9SF3	ENSG00000077147	-0,49	1,07E-02
CHN1	ENSG00000128656	0,69	1,09E-02
ABCE1	ENSG00000164163	0,34	1,13E-02
ROCK1	ENSG00000067900	0,42	1,14E-02
NDUFS5	ENSG00000168653	-0,55	1,15E-02
PPP1R16B	ENSG00000101445	-0,69	1,17E-02
SIDT2	ENSG00000149577	0,68	1,19E-02
MRPL40	ENSG00000185608	0,68	1,20E-02
CHAF1A	ENSG00000167670	-0,55	1,21E-02
RBMS1	ENSG00000153250	0,38	1,21E-02
GNPAT	ENSG00000116906	-0,59	1,22E-02
PIGH	ENSG00000100564	0,65	1,24E-02
H2AFX	ENSG00000188486	-0,48	1,24E-02
	ENSG00000198327	0,67	1,24E-02
SRPX	ENSG00000101955	0,49	1,25E-02
PUS7	ENSG00000091127	-0,63	1,25E-02
ACAT1	ENSG00000075239	-0,52	1,26E-02
TULP4	ENSG00000130338	0,64	1,28E-02
PPA1	ENSG00000180817	-0,47	1,29E-02
	ENSG00000124693	-0,65	1,32E-02
PTPRB	ENSG00000127329	0,34	1,33E-02
KIAA0930	ENSG00000100364	-0,68	1,35E-02
RPS14	ENSG00000164587	-0,43	1,36E-02
TSPAN18	ENSG00000157570	0,60	1,38E-02
BNIP3L	ENSG00000104765	0,61	1,40E-02
CREBBP	ENSG00000005339	0,50	1,41E-02
DEPDC1	ENSG00000024526	0,60	1,41E-02
ZFHX4	ENSG00000091656	-0,47	1,43E-02
CARD6	ENSG00000132357	-0,62	1,43E-02
INPP1	ENSG00000151689	-0,63	1,45E-02
GPT2	ENSG00000166123	-0,66	1,47E-02
KIAA1109	ENSG00000138688	0,51	1,48E-02
SETD4	ENSG00000185917	-0,65	1,50E-02
PCNP	ENSG00000081154	-0,54	1,51E-02
	ENSG00000262944	0,61	1,52E-02
B4GALT1	ENSG00000086062	0,47	1,53E-02
ZNF521	ENSG00000198795	0,49	1,53E-02
NUP85	ENSG00000125450	-0,63	1,56E-02
WDR12	ENSG00000138442	0,51	1,58E-02
PRCC	ENSG00000143294	-0,55	1,58E-02

LTBP1	ENSG00000049323	-0,47	1,59E-02
NIP7	ENSG00000132603	0,50	1,59E-02
FRYL	ENSG00000075539	-0,59	1,60E-02
	ENSG00000260539	0,59	1,61E-02
C19orf70	ENSG00000174917	-0,66	1,63E-02
PKN2	ENSG00000065243	0,44	1,63E-02
MYOF	ENSG00000138119	0,24	1,64E-02
TMEM201	ENSG00000188807	-0,65	1,64E-02
MYLK	ENSG00000065534	-0,62	1,64E-02
FAM220A	ENSG00000178397	0,65	1,65E-02
TUBE1	ENSG00000074935	-0,57	1,65E-02
PTP4A2	ENSG00000184007	-0,35	1,65E-02
ETV6	ENSG00000139083	-0,56	1,67E-02
RUSC1	ENSG00000160753	0,61	1,68E-02
NUDT16	ENSG00000198585	-0,56	1,69E-02
C19orf53	ENSG00000104979	-0,53	1,69E-02
TMX3	ENSG00000166479	0,35	1,69E-02
CUL4B	ENSG00000158290	0,43	1,70E-02
LACC1	ENSG00000179630	0,65	1,72E-02
ZCCHC11	ENSG00000134744	0,60	1,73E-02
RPA3	ENSG00000106399	-0,55	1,74E-02
ZNF383	ENSG00000188283	0,49	1,76E-02
CDC42	ENSG00000070831	0,32	1,76E-02
NRDC	ENSG00000078618	-0,41	1,76E-02
SLC2A10	ENSG00000197496	0,60	1,78E-02
RDH11	ENSG00000072042	0,38	1,79E-02
	ENSG00000260822	0,54	1,80E-02
CDK2AP1	ENSG00000111328	-0,54	1,82E-02
PES1	ENSG00000100029	-0,54	1,86E-02
ATP11A	ENSG00000068650	0,44	1,87E-02
LTV1	ENSG00000135521	-0,64	1,88E-02
UBL5	ENSG00000198258	-0,61	1,90E-02
GFOD1	ENSG00000145990	0,59	1,91E-02
PRDX3	ENSG00000165672	-0,39	1,92E-02
JADE2	ENSG00000043143	-0,64	1,92E-02
MAD2L1	ENSG00000164109	-0,58	1,94E-02
CCNL2	ENSG00000221978	-0,59	1,94E-02
OSTC	ENSG00000198856	-0,51	1,96E-02
ZNF185	ENSG00000147394	0,44	1,97E-02
ZNF768	ENSG00000169957	0,64	1,97E-02
ACIN1	ENSG00000100813	0,35	1,97E-02
BMPR2	ENSG00000204217	0,27	1,97E-02
FOXRED2	ENSG00000100350	0,57	1,98E-02
	ENSG00000077809	0,50	1,99E-02
ANTXR1	ENSG00000169604	-0,62	2,00E-02
TRAF7	ENSG00000131653	-0,37	2,01E-02

RAB4A	ENSG00000168118	-0,59	2,02E-02
EIF5	ENSG00000100664	0,38	2,03E-02
ZNF598	ENSG00000167962	-0,53	2,04E-02
RCOR1	ENSG00000089902	0,47	2,04E-02
OGFOD1	ENSG00000087263	-0,44	2,04E-02
TPD52L2	ENSG00000101150	-0,34	2,05E-02
SEL1L3	ENSG00000091490	0,38	2,06E-02
SPRYD3	ENSG00000167778	-0,61	2,07E-02
SNAP23	ENSG00000092531	-0,46	2,07E-02
RINT1	ENSG00000135249	-0,58	2,08E-02
PCDHGA11	ENSG00000253873	0,60	2,08E-02
DR1	ENSG00000117505	0,44	2,08E-02
MGRN1	ENSG00000102858	-0,55	2,09E-02
MRPL20	ENSG00000242485	0,50	2,14E-02
RER1	ENSG00000157916	-0,41	2,15E-02
SMYD4	ENSG00000186532	0,62	2,16E-02
RAB5A	ENSG00000144566	0,40	2,19E-02
FKBP15	ENSG00000119321	-0,50	2,20E-02
C18orf8	ENSG00000141452	0,61	2,22E-02
LRRC42	ENSG00000116212	-0,60	2,23E-02
ARL5B	ENSG00000165997	0,47	2,24E-02
GORASP2	ENSG00000115806	0,35	2,25E-02
ELF1	ENSG00000120690	0,43	2,27E-02
METTL6	ENSG00000206562	0,62	2,28E-02
TWISTNB	ENSG00000105849	0,43	2,28E-02
RAD23B	ENSG00000119318	0,31	2,28E-02
MLLT10	ENSG00000078403	0,59	2,29E-02
GLRX	ENSG00000173221	0,61	2,30E-02
SLC38A5	ENSG00000017483	0,52	2,30E-02
CHM	ENSG00000188419	0,53	2,32E-02
TFIP11	ENSG00000100109	0,56	2,33E-02
HDAC1	ENSG00000116478	-0,55	2,35E-02
TAF1B	ENSG00000115750	0,57	2,35E-02
KCMF1	ENSG00000176407	0,46	2,36E-02
GOT1	ENSG00000120053	-0,61	2,37E-02
CASP10	ENSG00000003400	0,58	2,37E-02
PRRC1	ENSG00000164244	0,40	2,38E-02
GPR135	ENSG00000181619	0,46	2,39E-02
DOLK	ENSG00000175283	-0,60	2,39E-02
PI4K2B	ENSG00000038210	-0,61	2,39E-02
SKIL	ENSG00000136603	-0,44	2,41E-02
RAP2A	ENSG00000125249	-0,56	2,44E-02
TFDP1	ENSG00000198176	-0,40	2,47E-02
ARL5A	ENSG00000162980	0,45	2,48E-02
BTF3	ENSG00000145741	0,29	2,49E-02
ZNF623	ENSG00000183309	0,61	2,49E-02

ITGA3	ENSG00000005884	0,38	2,49E-02
SPTBN5	ENSG00000137877	-0,61	2,51E-02
VTI1B	ENSG00000100568	-0,48	2,51E-02
CBX6	ENSG00000183741	-0,49	2,52E-02
STX4	ENSG00000103496	-0,57	2,53E-02
STOM	ENSG00000148175	0,29	2,53E-02
SLC45A3	ENSG00000158715	0,60	2,54E-02
KLC2	ENSG00000174996	-0,60	2,55E-02
UPF2	ENSG00000151461	0,45	2,55E-02
MPP4	ENSG00000082126	0,60	2,57E-02
ULK1	ENSG00000177169	-0,60	2,58E-02
TMEM237	ENSG00000155755	0,49	2,58E-02
OPA1	ENSG00000198836	0,37	2,65E-02
TES	ENSG00000135269	-0,57	2,65E-02
NUTF2	ENSG00000102898	0,44	2,65E-02
B3GNT5	ENSG00000176597	-0,49	2,66E-02
LMF2	ENSG00000100258	-0,58	2,66E-02
MIF	ENSG00000240972	0,60	2,69E-02
SKP1	ENSG00000113558	-0,42	2,70E-02
S1PR3	ENSG00000213694	-0,52	2,70E-02
UBQLN2	ENSG00000188021	0,55	2,70E-02
NOTCH2	ENSG00000134250	0,35	2,71E-02
FYCO1	ENSG00000163820	0,51	2,71E-02
MCM6	ENSG00000076003	-0,45	2,73E-02
BCR	ENSG00000186716	-0,49	2,73E-02
OAZ2	ENSG00000180304	-0,59	2,74E-02
CDV3	ENSG00000091527	0,24	2,74E-02
SEL1L	ENSG00000071537	0,36	2,77E-02
DPY30	ENSG00000162961	0,56	2,77E-02
ASXL2	ENSG00000143970	0,42	2,79E-02
GLOD4	ENSG00000167699	-0,55	2,79E-02
KLHL8	ENSG00000145332	-0,60	2,81E-02
RAF1	ENSG00000132155	0,45	2,83E-02
TTK	ENSG00000112742	-0,53	2,85E-02
TMEM69	ENSG00000159596	0,56	2,85E-02
USP37	ENSG00000135913	0,59	2,86E-02
OGT	ENSG00000147162	-0,40	2,87E-02
BIRC6	ENSG00000115760	0,31	2,87E-02
GPBP1L1	ENSG00000159592	0,45	2,87E-02
IGHMBP2	ENSG00000132740	-0,56	2,91E-02
	ENSG00000218283	0,60	2,91E-02
DNAJA2	ENSG00000069345	-0,51	2,92E-02
CPXM1	ENSG00000088882	0,48	2,93E-02
SLC30A1	ENSG00000170385	-0,55	2,93E-02
HIF1AN	ENSG00000166135	-0,51	2,94E-02
EIF6	ENSG00000242372	-0,45	2,94E-02

GCAT	ENSG00000100116	0,54	2,96E-02
MORF4L1	ENSG00000185787	-0,38	2,96E-02
HEG1	ENSG00000173706	0,38	2,97E-02
GFPT1	ENSG00000198380	-0,40	3,00E-02
NHSL2	ENSG00000204131	0,54	3,01E-02
TCAF1	ENSG00000198420	0,53	3,02E-02
STAM	ENSG00000136738	0,48	3,03E-02
TUBG1	ENSG00000131462	0,47	3,05E-02
MTMR12	ENSG00000150712	0,42	3,06E-02
UBE2D1	ENSG00000072401	-0,57	3,09E-02
UBA5	ENSG00000081307	0,49	3,10E-02
MAN2A2	ENSG00000196547	0,50	3,10E-02
RHOBTB3	ENSG00000164292	-0,44	3,11E-02
SMOX	ENSG00000088826	-0,59	3,14E-02
PRKRA	ENSG00000180228	-0,57	3,15E-02
MYCBP2	ENSG00000005810	0,42	3,15E-02
COX1	ENSG00000198804	0,24	3,17E-02
DNAJB5	ENSG00000137094	0,57	3,17E-02
ATP6V1G1	ENSG00000136888	-0,56	3,19E-02
SRRM1	ENSG00000133226	0,43	3,20E-02
DRAP1	ENSG00000175550	-0,51	3,22E-02
ABI3BP	ENSG00000154175	0,26	3,22E-02
UQCRC1	ENSG00000010256	-0,43	3,25E-02
NT5C2	ENSG00000076685	0,53	3,26E-02
LYRM7	ENSG00000186687	0,57	3,26E-02
HSF1	ENSG00000185122	0,43	3,27E-02
SMAD3	ENSG00000166949	-0,35	3,28E-02
MAP3K4	ENSG00000085511	0,50	3,29E-02
MTMR3	ENSG00000100330	-0,57	3,30E-02
MED14	ENSG00000180182	-0,43	3,30E-02
CASC4	ENSG00000166734	0,50	3,31E-02
MRC2	ENSG00000011028	-0,52	3,32E-02
BDNF	ENSG00000176697	-0,52	3,33E-02
HHIP-AS1	ENSG00000248890	0,42	3,33E-02
RUNX1	ENSG00000159216	-0,53	3,34E-02
FTSJ3	ENSG00000108592	-0,45	3,35E-02
ITGA10	ENSG00000143127	0,54	3,38E-02
SLC25A22	ENSG00000177542	-0,58	3,39E-02
SKA2	ENSG00000182628	0,44	3,39E-02
SSR2	ENSG00000163479	0,34	3,43E-02
MAPK1IP1L	ENSG00000168175	0,34	3,43E-02
ACP1	ENSG00000143727	-0,50	3,43E-02
SART3	ENSG00000075856	0,45	3,44E-02
NCR3LG1	ENSG00000188211	0,54	3,49E-02
SPOCK1	ENSG00000152377	0,21	3,49E-02
UNC13D	ENSG00000092929	-0,55	3,50E-02

MAN1A1	ENSG00000111885	0,54	3,50E-02
	ENSG00000135213	0,58	3,50E-02
ZFPL1	ENSG00000162300	-0,58	3,51E-02
FBXO42	ENSG00000037637	0,57	3,52E-02
ZNF260	ENSG00000254004	0,56	3,52E-02
TAP2	ENSG00000204267	0,50	3,53E-02
PPIA	ENSG00000196262	-0,25	3,58E-02
XRN1	ENSG00000114127	-0,49	3,58E-02
SUMO1	ENSG00000116030	-0,57	3,59E-02
FAM114A2	ENSG00000055147	0,57	3,60E-02
HMOX1	ENSG00000100292	0,45	3,61E-02
ITSN1	ENSG00000205726	0,38	3,62E-02
CSNK2A1	ENSG00000101266	0,31	3,62E-02
TMEM168	ENSG00000146802	-0,57	3,62E-02
DGKE	ENSG00000153933	0,57	3,63E-02
ARAF	ENSG00000078061	-0,50	3,64E-02
LGALS1	ENSG00000100097	-0,26	3,65E-02
CS	ENSG00000062485	0,34	3,65E-02
KIF21A	ENSG00000139116	-0,54	3,66E-02
C9orf64	ENSG00000165118	0,55	3,68E-02
ASAH1	ENSG00000104763	0,54	3,69E-02
METTL17	ENSG00000165792	-0,57	3,71E-02
EGFR	ENSG00000146648	0,53	3,74E-02
SNU13	ENSG00000100138	0,49	3,74E-02
MIB1	ENSG00000101752	0,38	3,74E-02
DDX42	ENSG00000198231	0,34	3,74E-02
UFD1L	ENSG00000070010	-0,50	3,76E-02
RASGRP2	ENSG00000068831	0,36	3,76E-02
NUP88	ENSG00000108559	0,37	3,76E-02
PBXIP1	ENSG00000163346	-0,47	3,78E-02
SLC29A1	ENSG00000112759	0,26	3,79E-02
SERPINB1	ENSG00000021355	0,56	3,81E-02
ZNF304	ENSG00000131845	0,54	3,81E-02
ZNF697	ENSG00000143067	-0,54	3,89E-02
SCRN3	ENSG00000144306	0,56	3,89E-02
PGRMC1	ENSG00000101856	0,44	3,92E-02
TM2D1	ENSG00000162604	-0,56	3,93E-02
CSNK1A1	ENSG00000113712	0,33	3,93E-02
BLVRA	ENSG00000106605	-0,55	3,94E-02
NUFIP2	ENSG00000108256	0,31	3,97E-02
NRDE2	ENSG00000119720	0,56	3,97E-02
GPR176	ENSG00000166073	-0,42	3,98E-02
BTF3L4	ENSG00000134717	0,49	3,99E-02
KLHL5	ENSG00000109790	0,42	3,99E-02
ND5	ENSG00000198786	0,25	3,99E-02
ZWINT	ENSG00000122952	-0,38	4,00E-02

UBE2H	ENSG00000186591	0,37	4,00E-02
TRIM25	ENSG00000121060	0,39	4,00E-02
DNAJC9	ENSG00000213551	-0,56	4,01E-02
EXOSC3	ENSG00000107371	-0,56	4,03E-02
GOLM1	ENSG00000135052	0,31	4,03E-02
DSCR3	ENSG00000157538	-0,50	4,03E-02
B4GAT1	ENSG00000174684	-0,56	4,03E-02
SLC31A1	ENSG00000136868	0,45	4,08E-02
BEX3	ENSG00000166681	-0,42	4,09E-02
C1GALT1	ENSG00000106392	-0,55	4,10E-02
REXO1	ENSG00000079313	0,53	4,11E-02
PHF13	ENSG00000116273	-0,55	4,11E-02
HERC4	ENSG00000148634	-0,51	4,12E-02
RIC8A	ENSG00000177963	-0,37	4,14E-02
MYO6	ENSG00000196586	0,35	4,14E-02
KIAA0907	ENSG00000132680	0,50	4,16E-02
PRPF39	ENSG00000185246	-0,56	4,16E-02
DONSON	ENSG00000159147	-0,56	4,16E-02
	ENSG00000250899	0,56	4,18E-02
SETD3	ENSG00000183576	-0,54	4,18E-02
MPP1	ENSG00000130830	0,56	4,18E-02
JPX	ENSG00000225470	0,54	4,19E-02
COPZ1	ENSG00000111481	-0,40	4,20E-02
EXOC1	ENSG00000090989	0,43	4,21E-02
PGAP3	ENSG00000161395	-0,39	4,22E-02
PRRC2B	ENSG00000130723	-0,26	4,22E-02
TRIAP1	ENSG00000170855	-0,50	4,22E-02
ANKFY1	ENSG00000185722	-0,44	4,23E-02
RAB22A	ENSG00000124209	0,40	4,24E-02
MERTK	ENSG00000153208	0,42	4,24E-02
EXOC5	ENSG00000070367	-0,39	4,24E-02
BCL2L12	ENSG00000126453	-0,54	4,24E-02
ICE1	ENSG00000164151	-0,50	4,24E-02
KIF18B	ENSG00000186185	0,46	4,25E-02
TMEM87B	ENSG00000153214	0,36	4,26E-02
	ENSG00000253352	0,31	4,27E-02
FAM172A	ENSG00000113391	0,54	4,28E-02
CDC123	ENSG00000151465	-0,37	4,31E-02
	ENSG00000237973	0,55	4,31E-02
SLC1A5	ENSG00000105281	-0,30	4,32E-02
LARP1B	ENSG00000138709	0,53	4,33E-02
ZNF283	ENSG00000167637	0,43	4,33E-02
ZBED4	ENSG00000100426	-0,54	4,33E-02
GGH	ENSG00000137563	-0,44	4,33E-02
GABARAPL2	ENSG00000034713	0,52	4,35E-02
KCNJ2	ENSG00000123700	0,55	4,35E-02

SLC5A6	ENSG00000138074	-0,53	4,37E-02
SSBP1	ENSG00000106028	-0,45	4,38E-02
CHTOP	ENSG00000160679	-0,54	4,40E-02
LRRC8E	ENSG00000171017	0,42	4,45E-02
XPC	ENSG00000154767	0,51	4,48E-02
MAML3	ENSG00000196782	0,55	4,51E-02
STARD7	ENSG00000084090	0,39	4,51E-02
MRPS33	ENSG00000090263	0,55	4,51E-02
ANLN	ENSG00000011426	0,21	4,52E-02
MIR568	ENSG00000259976	-0,34	4,52E-02
RCBTB1	ENSG00000136144	0,52	4,54E-02
ATP2A2	ENSG00000174437	0,24	4,54E-02
SGTB	ENSG00000197860	0,49	4,55E-02
TMPO	ENSG00000120802	0,29	4,56E-02
KLF10	ENSG00000155090	0,41	4,56E-02
MET	ENSG00000105976	0,28	4,56E-02
C8orf58	ENSG00000241852	-0,42	4,58E-02
DNAAF5	ENSG00000164818	-0,54	4,58E-02
RRP7A	ENSG00000189306	-0,47	4,59E-02
HCLS1	ENSG00000180353	0,41	4,59E-02
TMEM127	ENSG00000135956	-0,42	4,60E-02
C19orf43	ENSG00000123144	0,43	4,61E-02
IFI27	ENSG00000165949	-0,46	4,61E-02
EPAS1	ENSG00000116016	0,25	4,61E-02
ASNSD1	ENSG00000138381	-0,45	4,61E-02
RPL34	ENSG00000109475	-0,40	4,62E-02
PIP5K1C	ENSG00000186111	-0,38	4,63E-02
TMEM154	ENSG00000170006	0,42	4,63E-02
MEX3D	ENSG00000181588	-0,52	4,63E-02
HMBOX1	ENSG00000147421	0,52	4,63E-02
SPRY2	ENSG00000136158	-0,46	4,64E-02
RNASEH1-AS1	ENSG00000234171	0,53	4,64E-02
MICU1	ENSG00000107745	0,46	4,66E-02
KIFC3	ENSG00000140859	-0,47	4,67E-02
SFR1	ENSG00000156384	-0,48	4,68E-02
MED9	ENSG00000141026	0,52	4,69E-02
ALG13	ENSG00000101901	-0,54	4,69E-02
PHKA1	ENSG00000067177	0,54	4,69E-02
NUP43	ENSG00000120253	0,38	4,71E-02
TAX1BP1	ENSG00000106052	0,31	4,73E-02
GPR107	ENSG00000148358	0,41	4,73E-02
RPS18	ENSG00000231500	-0,24	4,73E-02
BRCC3	ENSG00000185515	0,47	4,76E-02
TMEM263	ENSG00000151135	-0,48	4,76E-02
POLR2A	ENSG00000181222	0,29	4,78E-02
CNTROB	ENSG00000170037	0,45	4,79E-02

SLC35C1	ENSG00000181830	-0,54	4,79E-02
C6orf203	ENSG00000130349	-0,49	4,83E-02
RAB1B	ENSG00000174903	0,37	4,87E-02
SLC16A5	ENSG00000170190	-0,37	4,87E-02
OTUD6B	ENSG00000155100	-0,48	4,87E-02
AIDA	ENSG00000186063	0,48	4,88E-02
SLC2A11	ENSG00000133460	-0,33	4,89E-02
PEX16	ENSG00000121680	0,53	4,90E-02
USP9X	ENSG00000124486	-0,35	4,90E-02
NAV3	ENSG00000067798	0,34	4,91E-02
LRRC40	ENSG00000066557	0,46	4,92E-02
NUP107	ENSG00000111581	0,44	4,94E-02
UBE2G1	ENSG00000132388	0,38	4,95E-02
PPP1R10	ENSG00000204569	-0,42	4,95E-02
CD109	ENSG00000156535	-0,39	4,96E-02
ABCF3	ENSG00000161204	-0,45	4,96E-02
GNG5	ENSG00000174021	0,34	4,97E-02

Supplemental Table 4.
Significant altered IPA-identified pathways in HUVECs cultured on non-targeting control versus on EMILIN1 deficient-ECM

**Ingenuity Canonical Pathways**	**-Log(p-value)**	**Ratio**	**z-score**
HGF Signaling	6,670	0,118	-0,577
GNRH Signaling	6,150	0,094	0
NGF Signaling	5,620	0,104	0,832
FLT3 Signaling in Hematopoietic Progenitor Cells	5,390	0,118	-0,302
UVC-Induced MAPK Signaling	5,120	0,163	-2,121
Regulation of eIF4 and p70S6K Signaling	5,030	0,086	-0,378
ErbB Signaling	4,920	0,106	-1,508
Aldosterone Signaling in Epithelial Cells	4,880	0,083	-1
Cholecystokinin/Gastrin-mediated Signaling	4,800	0,103	-1,508
B Cell Receptor Signaling	4,790	0,077	0,535
Endothelin-1 Signaling	4,740	0,077	-0,775
Opioid Signaling Pathway	4,690	0,069	-0,243
CCR3 Signaling in Eosinophils	4,510	0,088	-1,667
mTOR Signaling	4,460	0,073	-0,905
Molecular Mechanisms of Cancer	4,460	0,056	N/A
Phospholipase C Signaling	4,190	0,066	0,277
CXCR4 Signaling	4,160	0,076	-1,155
Huntington's Disease Signaling	4,070	0,064	-1,897
PAK Signaling	4,060	0,094	0,632
Role of Tissue Factor in Cancer	4,020	0,085	N/A
ERK/MAPK Signaling	3,950	0,069	-0,535
Germ Cell-Sertoli Cell Junction Signaling	3,950	0,073	N/A
LPS-stimulated MAPK Signaling	3,830	0,097	-1,667
Thrombin Signaling	3,820	0,067	-1,155
14-3-3-mediated Signaling	3,810	0,080	-1
Breast Cancer Regulation by Stathmin1	3,800	0,066	N/A
p70S6K Signaling	3,780	0,080	-1,508
CREB Signaling in Neurons	3,650	0,064	-0,302
Fc Epsilon RI Signaling	3,500	0,080	-2,53
Synaptic Long Term Potentiation	3,480	0,079	0
Neurotrophin/TRK Signaling	3,450	0,096	0,707
Renin-Angiotensin Signaling	3,420	0,078	-2,53
fMLP Signaling in Neutrophils	3,390	0,078	-0,632
UVB-Induced MAPK Signaling	3,340	0,106	-1,89
Erythropoietin Signaling	3,310	0,092	N/A
Renal Cell Carcinoma Signaling	3,280	0,091	-0,816
Sertoli Cell-Sertoli Cell Junction Signaling	3,270	0,065	N/A
GP6 Signaling Pathway	3,260	0,075	-2,53
EGF Signaling	3,260	0,103	-1,134
Prolactin Signaling	3,240	0,090	-1,89
Xenobiotic Metabolism Signaling	3,240	0,054	N/A
UVA-Induced MAPK Signaling	3,220	0,080	N/A
FGF Signaling	3,180	0,088	-0,707

Corticotropin Releasing Hormone Signaling	3,140	0,072	-1
TGF-β Signaling	3,120	0,086	-0,707
P2Y Purigenic Receptor Signaling Pathway	3,110	0,071	0
VEGF Family Ligand-Receptor Interactions	3,090	0,085	-1,89
Neuregulin Signaling	3,090	0,085	-1,134
Glioblastoma Multiforme Signaling	3,060	0,066	-0,302
Protein Kinase A Signaling	3,040	0,047	-0,775
ATM Signaling	2,970	0,082	-2,828
NRF2-mediated Oxidative Stress Response	2,970	0,060	-0,333
Acute Myeloid Leukemia Signaling	2,940	0,081	-0,378
ErbB4 Signaling	2,900	0,090	-1,89
IL-8 Signaling	2,890	0,059	-0,577
Hereditary Breast Cancer Signaling	2,890	0,067	N/A
GM-CSF Signaling	2,870	0,089	-1,134
Melanocyte Development and Pigmentation Signaling	2,800	0,077	0,707
GDNF Family Ligand-Receptor Interactions	2,770	0,085	-0,816
Cardiac Hypertrophy Signaling	2,730	0,054	1,155
Actin Nucleation by ARP-WASP Complex	2,720	0,097	0,816
Gαq Signaling	2,660	0,062	0
Macropinocytosis Signaling	2,620	0,081	-1,633
Integrin Signaling	2,620	0,055	0
Mouse Embryonic Stem Cell Pluripotency	2,600	0,071	-0,707
IGF-1 Signaling	2,600	0,071	-0,378
Production of Nitric Oxide and Reactive Oxygen Species in Macrophages	2,560	0,057	-0,905
Role of NFAT in Cardiac Hypertrophy	2,520	0,053	-1,508
Tec Kinase Signaling	2,490	0,059	-1,667
EIF2 Signaling	2,490	0,053	-0,816
Mitochondrial Dysfunction	2,470	0,059	N/A
Adrenomedullin signaling pathway	2,460	0,055	-1,667
α-Adrenergic Signaling	2,460	0,075	-1,633
NF-κB Activation by Viruses	2,460	0,075	-1,134
Fcγ Receptor-mediated Phagocytosis in Macrophages and Monocytes	2,460	0,075	-0,378
Gap Junction Signaling	2,440	0,055	N/A
HER-2 Signaling in Breast Cancer	2,430	0,075	N/A
Thrombopoietin Signaling	2,420	0,085	-1,633
Glioma Signaling	2,410	0,067	-1,89
Pancreatic Adenocarcinoma Signaling	2,410	0,067	-0,816
G Beta Gamma Signaling	2,410	0,067	-0,707
Systemic Lupus Erythematosus Signaling	2,400	0,052	N/A
Melatonin Signaling	2,390	0,083	-1
Sumoylation Pathway	2,380	0,073	-1,342
Parkinson's Signaling	2,380	0,188	N/A
Rac Signaling	2,350	0,065	1,134
Ephrin Receptor Signaling	2,330	0,056	1,667
Synaptic Long Term Depression	2,310	0,056	-1,265
Leukocyte Extravasation Signaling	2,280	0,052	-0,905

Role of MAPK Signaling in the Pathogenesis of Influenza	2,220	0,077	N/A
Prostate Cancer Signaling	2,220	0,068	N/A
Phagosome Formation	2,180	0,061	N/A
Interferon Signaling	2,180	0,111	N/A
Signaling by Rho Family GTPases	2,140	0,048	1,897
Colorectal Cancer Metastasis Signaling	2,110	0,047	-0,905
Non-Small Cell Lung Cancer Signaling	2,090	0,072	-1,342
Androgen Signaling	2,070	0,058	-0,447
Axonal Guidance Signaling	2,070	0,039	N/A
SAPK/JNK Signaling	2,060	0,064	-0,378
Growth Hormone Signaling	2,040	0,071	-0,816
Protein Ubiquitination Pathway	1,970	0,045	N/A
IL-3 Signaling	1,950	0,067	-1,633
Virus Entry via Endocytic Pathways	1,940	0,060	N/A
Telomerase Signaling	1,930	0,060	-1,89
Regulation of Actin-based Motility by Rho	1,930	0,067	2,449
Paxillin Signaling	1,890	0,059	-1,134
Role of JAK1, JAK2 and TYK2 in Interferon Signaling	1,870	0,125	N/A
Ovarian Cancer Signaling	1,850	0,053	-0,447
CCR5 Signaling in Macrophages	1,820	0,063	-2,236
CNTF Signaling	1,800	0,071	0,447
Apoptosis Signaling	1,800	0,063	0,816
Role of IL-17F in Allergic Inflammatory Airway Diseases	1,800	0,087	2
PTEN Signaling	1,780	0,056	-0,378
G-Protein Coupled Receptor Signaling	1,780	0,043	N/A
Antiproliferative Role of TOB in T Cell Signaling	1,780	0,115	N/A
ERK5 Signaling	1,750	0,069	1,342
D-myo-inositol (1,4,5)-Trisphosphate Biosynthesis	1,730	0,111	N/A
Natural Killer Cell Signaling	1,730	0,055	N/A
RANK Signaling in Osteoclasts	1,680	0,059	0
Cell Cycle: G2/M DNA Damage Checkpoint Regulation	1,680	0,080	N/A
Hypoxia Signaling in the Cardiovascular System	1,680	0,067	N/A
Myc Mediated Apoptosis Signaling	1,660	0,066	N/A
Chemokine Signaling	1,640	0,065	-0,447
Dopamine-DARPP32 Feedback in cAMP Signaling	1,640	0,049	0
CDK5 Signaling	1,630	0,057	1,342
CD27 Signaling in Lymphocytes	1,600	0,076	0
Cdc42 Signaling	1,600	0,048	1,134
Iron homeostasis signaling pathway	1,590	0,051	N/A
Role of BRCA1 in DNA Damage Response	1,570	0,063	0
Cyclins and Cell Cycle Regulation	1,550	0,062	N/A
BMP signaling pathway	1,530	0,061	-0,447
eNOS Signaling	1,530	0,047	-0,378
IL-15 Signaling	1,530	0,061	N/A
Choline Biosynthesis III	1,530	0,154	N/A
GPCR-Mediated Nutrient Sensing in Enteroendocrine Cells	1,510	0,054	-0,816

Chronic Myeloid Leukemia Signaling	1,510	0,054	N/A
Inhibition of Angiogenesis by TSP1	1,470	0,088	N/A
Neuropathic Pain Signaling In Dorsal Horn Neurons	1,460	0,052	-1,633
Regulation of IL-2 Expression in Activated and Anergic T Lymphocytes	1,460	0,058	N/A
Insulin Receptor Signaling	1,440	0,048	-0,378
HIPPO signaling	1,440	0,058	N/A
Phagosome Maturation	1,430	0,047	N/A
Melanoma Signaling	1,410	0,066	-1
Role of Macrophages, Fibroblasts and Endothelium in Rheumatoid Arthritis	1,410	0,038	N/A
JAK/Stat Signaling	1,400	0,056	-1,342
Epithelial Adherens Junction Signaling	1,400	0,047	N/A
p38 MAPK Signaling	1,380	0,050	0
Epoxysqualene Biosynthesis	1,380	0,500	N/A
L-cysteine Degradation III	1,380	0,500	N/A
Uridine-5'-phosphate Biosynthesis	1,380	0,500	N/A
IL-7 Signaling Pathway	1,370	0,055	-2,236
IL-17 Signaling	1,370	0,055	N/A
Activation of IRF by Cytosolic Pattern Recognition Receptors	1,360	0,064	0
Gustation Pathway	1,350	0,046	N/A
Factors Promoting Cardiogenesis in Vertebrates	1,350	0,054	N/A
PEDF Signaling	1,330	0,054	-1,342
Pyridoxal 5'-phosphate Salvage Pathway	1,320	0,062	-1
RAR Activation	1,320	0,042	N/A
Calcium-induced T Lymphocyte Apoptosis	1,300	0,061	-2
Role of NFAT in Regulation of the Immune Response	1,300	0,042	-0,816

N/A = Not applicable. IPA did not predict a z-score.

Supplemental Table 5.

Significant altered IPA-identified pathways in HUVECs cultured on non-targeting control versus on FBN1-deficient ECM

Ingenuity Canonical Pathways	-Log(p-value)	Ratio	z-score
Hypoxia Signaling in the Cardiovascular System	3,780	0,107	N/A
UVC-Induced MAPK Signaling	3,290	0,122	-0,816
TGF-β Signaling	3,140	0,086	-0,378
Nucleotide Excision Repair Pathway	3,130	0,143	N/A
GNRH Signaling	3,020	0,064	1,508
Heme Degradation	2,600	0,500	N/A
HGF Signaling	2,460	0,067	1,134
Caveolar-mediated Endocytosis Signaling	2,440	0,085	N/A
Ephrin Receptor Signaling	2,350	0,056	2,53
PTEN Signaling	2,330	0,064	-2,121
Thrombin Signaling	2,330	0,052	1
Role of Tissue Factor in Cancer	2,230	0,062	N/A
ErbB Signaling	2,210	0,067	0,378
CCR3 Signaling in Eosinophils	2,110	0,059	-0,447
Neurotrophin/TRK Signaling	2,110	0,072	0,816
Cdc42 Signaling	2,070	0,054	1,633
Actin Nucleation by ARP-WASP Complex	2,030	0,081	1,342
Macropinocytosis Signaling	2,010	0,069	-0,447
Regulation of Actin-based Motility by Rho	1,950	0,067	1,633
Clathrin-mediated Endocytosis Signaling	1,930	0,048	N/A
Pancreatic Adenocarcinoma Signaling	1,890	0,058	0,447
Breast Cancer Regulation by Stathmin1	1,870	0,047	N/A
Epithelial Adherens Junction Signaling	1,870	0,053	N/A
Neuregulin Signaling	1,860	0,064	0,447
EGF Signaling	1,860	0,074	1,342
Rac Signaling	1,830	0,057	0,816
Apoptosis Signaling	1,820	0,063	0,816
NGF Signaling	1,800	0,056	1,633
Huntington's Disease Signaling	1,790	0,044	0
Synaptic Long Term Potentiation	1,780	0,056	1,134
Melatonin Signaling	1,770	0,069	N/A
Ephrin B Signaling	1,740	0,069	2
D-myo-inositol (1,4,5)-Trisphosphate Biosynthesis	1,740	0,111	N/A
Glutamate Removal from Folates	1,680	1,000	N/A
Dopamine-DARPP32 Feedback in cAMP Signaling	1,660	0,049	1,342
Chemokine Signaling	1,650	0,065	0,447
CDK5 Signaling	1,650	0,057	0,447
Protein Ubiquitination Pathway	1,630	0,042	N/A
NRF2-mediated Oxidative Stress Response	1,620	0,045	0
Cholecystokinin/Gastrin-mediated Signaling	1,610	0,056	0,816
PAK Signaling	1,610	0,056	1,633
Iron homeostasis signaling pathway	1,610	0,051	N/A
IL-8 Signaling	1,570	0,044	1

BMP signaling pathway	1,550	0,061	0,447
Choline Biosynthesis III	1,540	0,154	N/A
Mouse Embryonic Stem Cell Pluripotency	1,530	0,054	0
Axonal Guidance Signaling	1,510	0,035	N/A
DNA Methylation and Transcriptional Repression Signaling	1,480	0,088	N/A
Phenylalanine Degradation IV (Mammalian, via Side Chain)	1,480	0,143	N/A
Renal Cell Carcinoma Signaling	1,430	0,057	1
Hereditary Breast Cancer Signaling	1,420	0,047	N/A
Signaling by Rho Family GTPases	1,410	0,040	2,121
Interferon Signaling	1,410	0,083	N/A
Glioma Signaling	1,400	0,050	0,447
G Beta Gamma Signaling	1,400	0,050	0,816
Integrin Signaling	1,390	0,041	1
Dolichol and Dolichyl Phosphate Biosynthesis	1,380	0,500	N/A
Alanine Degradation III	1,380	0,500	N/A
Alanine Biosynthesis II	1,380	0,500	N/A
L-cysteine Degradation III	1,380	0,500	N/A
Threonine Degradation II	1,380	0,500	N/A
NF-κB Signaling	1,370	0,043	1,414
PEDF Signaling	1,350	0,054	-1,342
LPS-stimulated MAPK Signaling	1,350	0,054	-0,447
FLT3 Signaling in Hematopoietic Progenitor Cells	1,350	0,054	0,447
Notch Signaling	1,350	0,079	N/A
Role of NFAT in Cardiac Hypertrophy	1,330	0,040	0,378
UVB-Induced MAPK Signaling	1,310	0,061	0
Oncostatin M Signaling	1,300	0,075	N/A

N/A = Not applicable. IPA did not predict a z-score.

Supplemental Table 6.
Different pooled samples of fetal and mature human renal artery

	Fetal	Mature
**Pool 1**	3 donors (19, 21 and 22 weeks)	2 donors (48 and 44 years)
**Pool 2**	3 donors (16, 20 and 22 weeks)	2 donors (25 and 45 years)
**Pool 3**	2 donors (17 and 21 weeks)	2 donors (60 and 63 years)

Supplemental Table 7.
siRNA sequences for knockdown experiments

Target gene	Target sequence
siSham (Non-targeting control)	UGGUUUACAUGUCGACUAA
	UGGUUUACAUGUUGUGUGA
	UGGUUUACAUGUUUUCUGA
	UGGUUUACAUGUUUUCCUA
siEMILIN1	AGGAUGGAGUGGAGACAUA
	AUACUAAACGAUCGAGGAA
	GGGCAACCAAGGACCGUAU
	CAGAGAGUGAAGAGCGCUU
siFBN1	CGAAUGAGCUACUGUUAUG
	CAAUACAGAUGGUUCCUAU
	GCGAGUGUCCCUUUGGUUA
	CGAGUUGGCUGUGUUGAUA

Supplemental Table 8.
Primer sequences used for quantitative PCR

Target gene	Sense primer sequence	Antisense primer sequence
B-actin	TCCCTGGAGAAGAGCTACGA	AGCACTGTGTTGGCGTACAG
EMILIN1	CCACGCTGGAGGGATTACAAG	TCAGCCGTAGTGTGAACTCTG
FBN1	TTTAGCGTCCTACACGAGCC	CCATCCAGGGCAACAGTAAGC
